# Copper Depletion Strongly Enhances Ferroptosis via Mitochondrial Perturbation and Reduction in Antioxidative Mechanisms

**DOI:** 10.3390/antiox11112084

**Published:** 2022-10-22

**Authors:** Fan Li, Xiaojing Wu, Hongli Liu, Mengqi Liu, Zhengkai Yue, Zhenyu Wu, Lei Liu, Fuchang Li

**Affiliations:** 1 Shandong Provincial Key Laboratory of Animal Biotechnology and Disease Control and Prevention, Department of Animal Science, Shandong Agricultural University, Tai’an 271018, China; 2 Hebei Key Laboratory of Specialty Animal Germplasm Resources Exploration and Innovation, Department of Animal Science and Technology, Hebei Normal University of Science and Technology, Qinhuangdao 066004, China; 3 College of Animal Science and Technology, Henan Agricultural University, Zhengzhou 450002, China

**Keywords:** ferroptosis, copper, oxidative stress, antioxidant, glutathione

## Abstract

Copper serves as a co-factor for a host of metalloenzymes, particularly cytochrome c oxidase (COX). Although it is known that impaired COX function can lead to the excessive accumulation of reactive oxygen species (ROS), the mechanisms underlying how copper depletion leads to cell damage are poorly understood. Here, we have investigated the role of copper depletion during ferroptosis. The bathocuproinedisulfonic (BCS) treatment depolarized the mitochondrial membrane potential, increased the total cellular ROS levels, stimulated oxidative stress, and reduced the glutathione levels. Moreover, the depletion of copper limited the protein expression of glutathione peroxidase 4 (GPX4), which is the only enzyme that is known to prevent lipid peroxidation. Furthermore, we found that copper depletion decreased the sensitivity of the dermal papilla cells (DPCs) to erastin (an inducer of ferroptosis), and the ferroptosis inhibitor ferrostatin-1 (Fer-1) partially prevented BCS-mediated cell death. Overall, these findings establish a direct link between copper and ferroptosis; BCS-mediated copper depletion strongly enhances ferroptosis via mitochondrial perturbation and a reduction in antioxidative mechanisms.

## 1. Introduction

Cell metabolism is precisely controlled for the maintenance of intracellular homeostasis [1,2] and ATP is the major energy source for cell growth [3]. The progressive oxidation of ingested nutrients provides electrons, and the continuous transfer of electrons in the ETC allows the cells to produce ATP continuously and steadily [4]. However, mitochondrial ATP production is accompanied by the generation of reactive oxygen species (ROS), which is the normal by-product of aerobic metabolism. The excessive ROS accumulation leads to oxidative stress and can, in combination with free ferrous iron (Fe^2+^), lead to ferroptosis [5]. Accumulating evidence indicates an intimate link between metabolism and ferroptosis via energy-stress-mediated AMPK (AMP-activated protein kinase) activation [6]. In fact, AMPK is a critical sensor of the cellular energy status [3]. Once it is activated, AMPK reprograms the metabolism to promote the catabolic processes and inhibit the anabolic processes [7,8]. Several studies have demonstrated that copper depletion can induce the activation of AMPK [7,8,9]. In the absence of copper atoms, the cytochrome c oxidase (COX) fails to assemble, and electron transport is suppressed, which ultimately leads to the decrease in mitochondrial ATP production and the activation of AMPK [10,11]. In addition, the inhibition of electron transport can lead to excessive ROS accumulation [12].

Ferroptosis is induced by the overproduction of phospholipid hydroperoxides, which is different from apoptosis, necrosis, and autophagy [6]. The hallmark event of ferroptosis is the inactivation of the glutathione peroxidase 4 (GPX4), the subsequent accumulation of ROS [13,14,15] and, in combination with free ferrous iron (Fe^2+^), the increased damage to the membrane lipids [16]. The cells regulate intracellular redox homeostasis through a complex endogenous antioxidant defense network that includes antioxidant enzymes (e.g., superoxide dismutase (SOD) 1, 2, and 3 and glutathione peroxidase (GSH-Px)), non-enzymatic compounds (e.g., glutathione and proteins), and low-molecular-weight scavengers (e.g., uric acid and lipoic acid) [17]. Superoxide dismutase (SOD) is the major antioxidant defense system in mammals, which consist of the following three isoforms: SOD1 (Cu/ZnSOD), which is present in the cytosol and the intermembrane space of the mitochondria, SOD2 (MnSOD), which is present in the mitochondria, and the extracellular SOD3 (Cu/ZnSOD) [18]. GPX4 uses reduced glutathione (composed of glutamate, glycine, and cysteine) to convert phospholipid hydroperoxides to lipid alcohols and inhibits ferroptosis [19]. Cysteine is the rate-limiting precursor for the synthesis of reduced glutathione [20]. The cells obtain cysteine through System Xc-, which transfers cystine into the cells and transfers glutamate out of cells in a 1:1 ratio at the same time, and higher contents of glutamate inhibit System Xc- transport, resulting in an increased depletion of cysteine [20]. Although cystine/glutamate can be interconverted intracellularly, the uptake of cystine from extracellular to intracellular is the dominant manner for cells to obtain cysteine [21]. Yang et al. reported that a decreased uptake of cystine caused a reduced synthesis of GSH, leading to the inhibition of GPX4 and the loss of intracellular antioxidant capacity, which eventually lead to ferroptosis [22].

To date, extensive studies have indicated a link between metabolism and ferroptosis [6,13,14]; however, the role of copper in the regulation of ferroptosis is unclear. Here, we report that bathocuproinedisulfonic (BCS)-mediated copper depletion strongly enhances ferroptosis in the dermal papilla cells (DPCs). We have found that BCS-mediated copper depletion resulted in metabolic reprogramming, which was characterized by a depletion in GSH and GPX4 inactivation. In addition, our results have shown that copper depletion decreased the sensitivity of DPCs to erastin (an inducer of ferroptosis), while the ferroptosis inhibitor ferrostatin-1 (Fer-1) partially prevented BCS-mediated cell death. Together, these data identify an essential role of copper in ferroptosis and contribute to an emerging paradigm of the involvement of metals in cell processes.

## 2. Materials and Methods

### 2.1. Materials

Bathocuproinedisulfonic acid (BCS) (B1125, Sigma, St. Louis, OH, USA) and ferrostatin-1 (Fer-1) (SML0583, Sigma, OH, USA) were purchased from Sigma-Aldrich. Erastin was purchased from Beyotime (SC0224, Beyotime, Shanghai, China). The graphical abstract was drawn by Figdraw (www.figdraw.com, accessed on 9 September 2022).

### 2.2. Cells

Dermal papilla cells (DPCs) from Rex rabbits were kindly provided by Professor Xin Sheng Wu (College of Animal Science and Technology, Yangzhou University, Yangzhou, China) and were identified as previously described. The results showed that the isolated DPCs had high alkaline phosphatase activity and the marker proteins α smooth muscle actin (α-SMA) and versican (Vim) were positive [23].

### 2.3. Cell Culture

The DPCs were cultured at 37 °C in an incubator with 5% CO_2_. Before each experiment, the DPCs were serum-starved overnight in Dulbecco’s modified Eagle’s medium (DMEM; Thermo Fisher, Carlsbad, CA, USA) without fetal bovine serum.

### 2.4. Measurement of ATP Content

The ATP content was determined using an ATP detection kit (S0026, Beyotime, Shanghai, China). Fluorescence was measured with a fluorescence microplate reader (BioTek Instruments, Winooski, VT, USA). The ATP content was normalized to the protein content.

### 2.5. Cytochrome c Oxidase Activity

The COX activity was measured using a mitochondrial respiratory chain complex IV activity kit (BC0945; Beijing Solarbio Science & Technology Co., Ltd., Beijing, China). The difference in activity between the control and the bathocuproinedisulfonic acid (BCS) treatment groups was determined at 550 nm with a Varioskan LUX microplate reader (Thermo Fisher, Carlsbad, CA, USA) using the 1st and 15th min readings. Complex IV activity was normalized to the protein content.

### 2.6. Assay for Mitochondrial Membrane Potential (MMP)

The MMP was determined with an MMP detection kit (C2006, Beyotime, Shanghai, China). The chemiluminescence signal was detected using a fluorescence microplate reader (BioTek Instruments, Carlsbad, CA, USA). CCCP (a membrane uncoupling chemical) was used as a positive control. The results were presented as relative fluorescence intensity and were normalized to that of the control group.

### 2.7. Determination of Total Cellular ROS Concentrations

The total cellular ROS levels were measured using a fluorescent molecular probe (20,70-dichlorodihydrofluorescein diacetate (DCFH-DA); S0033S, Beyotime, Shanghai, China). Rosup, which is a ROS inducer, served as a positive control. The results were presented as fluorescence intensity and were normalized to that of the control group.

### 2.8. SOD1 Activity

The intracellular SOD1 activity was determined using a SOD1 and SOD2 assay kit (S0103, Beyotime, Shanghai, China). The absorbance was detected at 450 nm and the activity of SOD1 was normalized to the protein content.

### 2.9. Measurement of GSH-Px Activity

The intracellular GSH-Px activity was determined using a GSH-Px assay kit (S0057S, Beyotime, Shanghai, China). The absorbance was detected at 412 nm and the activity of GSH-Px was normalized to the protein content.

### 2.10. Detection of Intracellular GSH and GSSG Levels

The intracellular levels of GSH and GSSG were determined with a respective assay kit (S0053S, Beyotime, Shanghai, China). T-GSH and GSSG contents were detected, and the absorbance was measured at 412 nm. 

### 2.11. Determination of MDA Concentrations

The intracellular levels of MDA were determined with an assay kit (BC0025, Solarbio, Beijing, China). MDA content was detected, and the absorbance was measured at 532 nm.

### 2.12. Determination of Fe^2+^ Concentrations

The intracellular levels of Fe^2+^ were determined with an assay kit (BC4355, Solarbio, Beijing, China). Fe^2+^ content was detected, and the absorbance was measured at 520 nm.

### 2.13. Determination of Glutamic Concentrations

The intracellular levels of glutamic were determined with an assay kit (BC1580, Solarbio, Beijing, China). Glutamic content was detected, and the absorbance was measured at 340 nm.

### 2.14. Determination of Cysteine Concentrations

The intracellular levels of cysteine were determined with an assay kit (BC0185, Solarbio, Beijing, China). Cysteine content was detected, and the absorbance was measured at 600 nm.

### 2.15. Determination of Lipid Peroxide Concentrations

The intracellular levels of lipid peroxide were determined with an assay kit (BC5240, Solarbio, Beijing, China). Cysteine content was detected, and the absorbance was measured at 532 nm.

### 2.16. Determination of Protein Carbonylation Concentrations

The intracellular levels of cysteine were determined with an assay kit (BC1270, Solarbio, Beijing, China). Cysteine content was detected, and the absorbance was measured at 370 nm.

### 2.17. Measurement of Copper Contents

The BCS-treated DPCs (2 × 10^7^) were digested with 50% HNO_3_ + 0.01% digitonin at 65 °C and the copper content was measured against copper standards using Analytik Jena novAA 400P (Jena, Germany).

### 2.18. RNA Isolation and Analysis

Extraction of total RNA and reverse-transcription quantitative PCR were performed as described previously [24,25], and the total RNA was extracted by the Trizol (Thermo Fisher, Carlsbad, CA, USA) method. The RNA concentration was measured on a DU 640 nucleic acid spectrophotometer (Beckman Coulter, Inc., 250 S. Kraemer Boulevard Brea, CA, USA). The quality of extracted RNA was detected with agarose gel electrophoresis. The reverse-transcription reactions (20 μL) contained 1000 ng of the total RNA, 4 μL 5 ×Evo M-MLVRT master mix (supplied by the Accurate Biotechnology Co., Ltd., Hunan, China). Real-time PCR analysis was carried out with an Applied Biosystems 7500 real-time PCR system (Applied Biosystems, Foster, CA, USA). Each RT reaction served as a template in a 20 μL PCR containing 0.2 mol/L of each primer and SYBR Green master mix (Vazyme, Nanjing, China). The gene for normalization was GAPDH (glyceraldehyde-3-phosphate dehydrogenase), and the results of relative mRNA quantification were verified using β-actin levels. The mRNA expression was analyzed using the 2^−ΔΔCT^ method [24,25]. Primer sequences are shown in Supplementary Table S1.

### 2.19. Western Blot

The total protein was extracted using a radioimmunoprecipitation assay (RIPA) lysis buffer (Solarbio, Beijing, China) with an addition of PMSF and protease inhibitor. The proteins were separated on a 7.5–10% SDS polyacrylamide gel electrophoresis, transferred onto PVDF membranes at 200 mA at 4 °C, and enclosed in closing solution (New Cell Molecular Biotechnology Co., Ltd., Shanghai, China) for 120 min. Protein detection was performed using the enhanced chemiluminescence detection reagents (Cat# P10200, New Cell Molecular Biotechnology Co., Ltd., Shanghai, China). GAPDH (glyceraldehyde-3-phosphate dehydrogenase) was used as a loading control to quantify the other protein levels. Western blots were developed and quantified with a BioSpectrum 810 Imaging System using VisionWorksLS 7.1 software (UVP LLC, Upland, CA, USA). The standard markers for protein molecular masses were supplied by Thermo Fisher (Cat# 26617, Thermo Fisher, Carlsbad, CA, USA). The membranes were probed with the following required antibodies: OXPHOS antibody cocktail (Cat# ab110413, abcam), GPX4 (Cat# ab125066, abcam) GAPDH (Cat# ab9485, abcam), AMPK (Cat# 10929-2-AP, Proteintech), and p-AMPK (thr172) (Cat#50081, CST). The horseradish peroxidase (HRP)-conjugated goat anti-rabbit IgG and goat anti-mouse IgG antibody were supplied by Beyotime (Beyotime, Shanghai, China).

### 2.20. Liquid Chromatography–Mass Spectrometry (LC–MS) Analysis

Ultra-high performance liquid chromatography–tandem mass spectrometry (UHPLC–MS/MS) was conducted by Shanghai Biotree Biomedical Technology Co., Ltd. (Shanghai, China). A total of 8905 peaks were detected, with 845 metabolites remaining after relative standard deviation denoising. The missing values were then filled in with half of the minimum values. Additionally, the LC–MS data were subjected to internal standard normalization. The final dataset containing the information for the peak number, sample name, and normalized peak area was imported into the SIMCA16.0.2 software package (Sartorius Stedim Data Analytics AB, Umea, Sweden) for multivariate analysis. The data were scaled and logarithmically transformed to minimize the impact of both the noise and high variance of the variables. Subsequently, principal component analysis (PCA) was carried out to visualize the sample distribution and grouping. The 95% confidence interval in the PCA score plot was used as the threshold to identify potential outliers in the dataset. To visualize group separation and identify significantly changed metabolites, supervised orthogonal projections to latent structures-discriminate analysis (OPLS-DA) was applied, after which a 7-fold cross validation was performed to calculate the R2 and Q2 values. To assess the robustness and predictive ability of the OPLS-DA model, a permutation test (200 times) was also performed. Afterwards, the R2 and Q2 intercept values were obtained. Here, the Q2 intercept value (the lower the better) represents the robustness of the model, the risk of overfitting, and the reliability of the model.

The value of the variable importance in the projection (VIP) score of the first principal component in the OPLS-DA analysis, which summarizes the contribution of each variable to the model, was also obtained. Metabolites with a VIP score >1 and *p* < 0.05 (Student’s t-test) were considered to have significantly changed abundance. The KEGG (http://www.genome.jp/kegg/, accessed 10 August 2022) and MetaboAnalyst (http://www.metaboanalyst.ca/, accessed 10 August 2022) databases were used for pathway enrichment analysis.

### 2.21. Statistical Analysis

The data were presented as the means ± SD. Before analysis, all data were examined for the homogeneity and normal distribution plots of variances among the treatments by using UNIVARIATE procedure. Data from more than two groups were analyzed by analysis of variance (ANOVA), followed by Tukey’s HSD or Dunnett’s multiple comparisons. The data from two groups were analyzed by Student’s t-test. All Statistical analyses were performed using SAS statistical software (SAS version 8.1, Cary, NC, USA). Differences were considered to be significant at *p* < 0.05.

## 3. Results

### 3.1. BCS Treatment Depleted Intracellular Copper Concentrations

**Figure 1:**
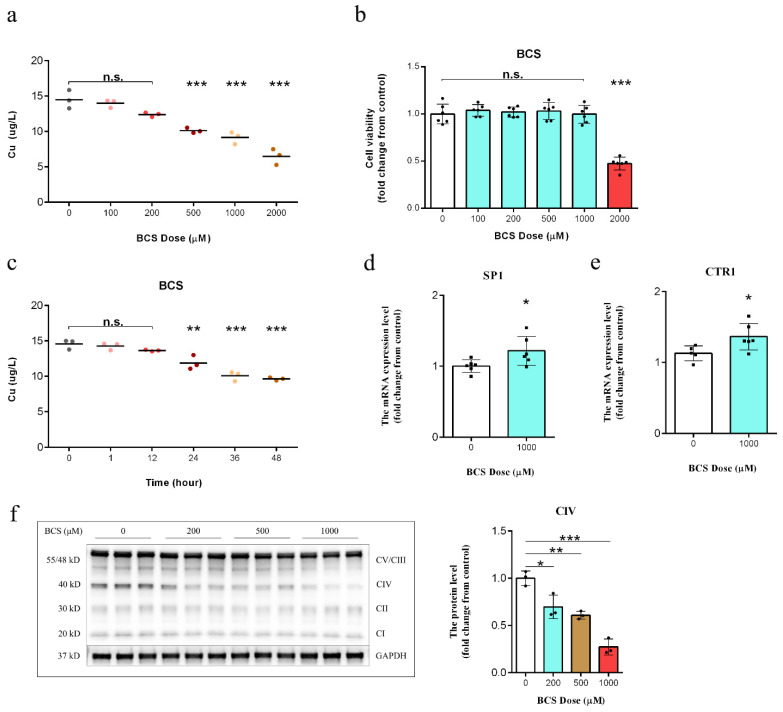
(**a**) Measurement of intracellular copper levels in dermal papilla cells (DPCs) after 72 h of treatment (*n* = 3/group). n.s. no significance; ** *p* < 0.01; *** *p* < 0.001. (**b**) A cell counting kit-8 (CCK-8) assay was used to assess the effect of different bathocuproinedisulfonic acid (BCS) concentrations on DPC viability after 72 h of treatment (*n* = 6/group). n.s. no significance; *** *p* < 0.001. (**c**) Measurement of intracellular copper levels in BCS-treated (1000 µM) DPCs (*n* = 3/group). n.s. no significance; *** *p* < 0.001. (**d**,**e**) qPCR analysis of the transcript levels of the SP1 (encoding transcription factor Sp1) and CTR1 (encoding a high-affinity copper transporter) genes in the control and BCS-treated (1000 µM) DPCs after 72 h of treatment (*n* = 6/group). * *p* < 0.05. (**f**) Western blot analysis of the CIV levels in DPCs following 72 h of BCS treatment (0, 200, 500, and 1000 µM) (*n* = 3/group). * *p* < 0.05, ** *p* < 0.01; *** *p* < 0.001.

We treated the DPCs with multiple concentrations of BCS in order to identify the lowest dose that could effectively deplete the intracellular copper levels. The BCS treatment led to a dose-dependent reduction in the levels of intracellular copper (Figure 1a). After 72 h of BCS (1000 µM), the treatment efficiently depleted the intracellular copper levels without affecting the cell viability (Figure 1b,c). The copper transporter 1 (CTR1) is a plasma membrane protein that regulates the cellular uptake of copper [26]. Sp1 transcription factor (SP1) is a transcription factor and the zinc finger domain of SP1 functions as a sensor of copper, which recognizes the DNA promoter sequence of CTR1 and regulates its mRNA level in response to intracellular copper concentration variations [27]. The increases in the mRNA levels of the SP1 and the CTR1 further confirmed the depletion of the intracellular copper with the BCS treatment (Figure 1d,e). The catalytic core of COX is formed by three mitochondria-encoded subunits (MTCO1, MTCO2, and MTCO3). In the absence of copper, both of the subunits are rapidly degraded, and the COX holoenzyme fails to assemble and function [28]. As expected, the protein level of MTCO1 was also found to be significantly reduced (Figure 1f).

### 3.2. Copper Depletion Impaired Mitochondrial Complex IV

**Figure 2:**
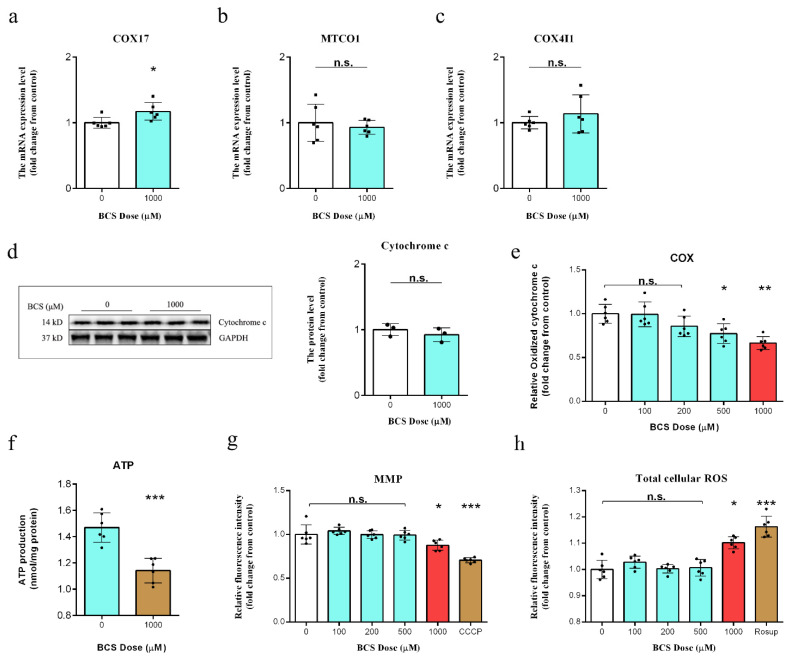
(**a**–**c**) qPCR analysis of the transcript levels of the COX17, MTCO1, and COX4I1 genes in the control and bathocuproinedisulfonic acid (BCS)-treated (1000 µM) dermal papilla cells (DPCs) after 72 h of treatment (*n* = 6/group). n.s. no significance; * *p* < 0.05. (**d**) The protein expression levels of cytochrome c with and without BCS treatment (1000 µM) (*n* = 3/group). n.s. no significance. (**e**) Cytochrome c oxidase activity measured after 72 h of treatment with BCS (*n* = 6/group). n.s. no significance; * *p* < 0.05; ** *p* < 0.01. (**f**) ATP production after 72 h of treatment with BCS (1000 µM) (*n* = 6/group). *** *p* < 0.001. (**g**) Measurement of mitochondrial membrane potential (MMP) in DPCs after 72 h of treatment with BCS (1000 µM) (*n* = 6/group). n.s. no significance; * *p* < 0.05; *** *p* < 0.001. (**h**) Measurement of total cellular ROS levels in DPCs after 72 h of treatment with bathocuproinedisulfonic acid (BCS) (1000 µM) (*n* = 6/group). n.s. no significance; * *p* < 0.05; *** *p* < 0.001.

As shown in Figure 2a, the mRNA levels of COX17 (encoding cytochrome c oxidase copper chaperone) were increased, which suggested that there are reduced copper concentrations in the mitochondria. As expected, the copper depletion did not affect the mRNA levels of either the MTCO1 (encoding cytochrome c oxidase subunit 1) (Figure 2b) or the COX4I1 (encoding cytochrome c oxidase subunit 4I1) (Figure 2c). The BCS-mediated copper depletion did not affect the protein level of cytochrome c (Figure 2d) but resulted in a significant reduction in the oxidation of cytochrome c (Figure 2e) and ATP production (Figure 2f), which indicated BCS-mediated inactivation of the mitochondrial COX rather than a loss of cytochrome c as the primary metabolic defect in the DPCs. Additionally, the BCS-mediated copper depletion resulted in a decrease in the MMP and an increase in the total cellular ROS generation (Figure 2g,h). 

### 3.3. Copper Depletion Impaired Antioxidant Capacity in DPCs

**Figure 3:**
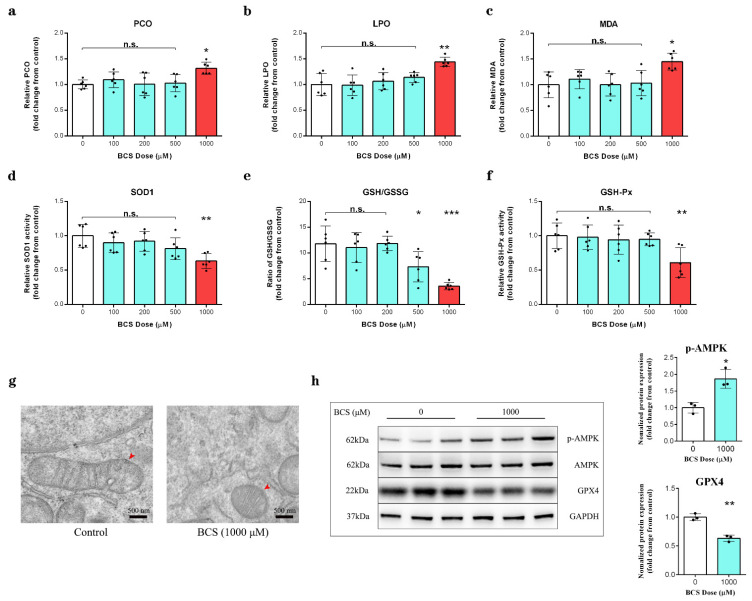
(**a**–**c**) Measurement of protein carbonylation, LPO, and MDA contents in DPCs after 72 h of treatment with BCS (1000 µM) (*n* = 6/group). n.s. no significance; * *p* < 0.05; ** *p* < 0.01. (**d**) Measurement of SOD1 activity in DPCs after 72 h of treatment with BCS (1000 µM) (*n* = 6/group). n.s. no significance; ** *p* < 0.01. (**e**) The GSH/GSSG ratio in DPCs was determined after 72 h of treatment with BCS (1000 µM) (*n* = 6/group). n.s. no significance; * *p* < 0.05; *** *p* < 0.001. (**f**) GSH-Px activity in DPCs was measured after 72 h of treatment with BCS (1000 µM) (*n* = 6/group). n.s. no significance; ** *p* < 0.01. (**g**) The structure of mitochondria as determined by transmission electron microscopy (×50,000), red arrows indicate normal/folded and perturbed cristae morphology in DPCs. Scale bar, 500 nm. (**h**) Western blot analysis of the protein levels of phosphorylated (p)-AMPK (Thr172) and GPX4 in the control and BCS-treated (1000 µM) DPCs (*n* = 3/group). n.s. no significance; * *p* < 0.05; ** *p* < 0.01.

The copper depletion led to a significant increase in protein carbonylation, lipid peroxide (LPO) and malondialdehyde (MDA) contents (Figure 3a–c), a significant reduction in SOD1 activity (Figure 3d), a significant decrease in the glutathione/oxidized glutathione (GSH/GSSH) ratio (Figure 3e), and the inhibition of GSH-Px activity (Figure 3f). Transmission electron microscopy (TEM) revealed that the DPCs that were treated with BCS exhibited shrunken mitochondria with a decreased number of cristae morphology (Figure 3g). Moreover, we observed that the BCS-mediated copper depletion in the DPCs increased the levels of AMPK phosphorylation at Thr172 and decreased the protein level of GPX4(Figure 3h).

### 3.4. UHPLC–MS/MS Analysis

**Figure 4:**
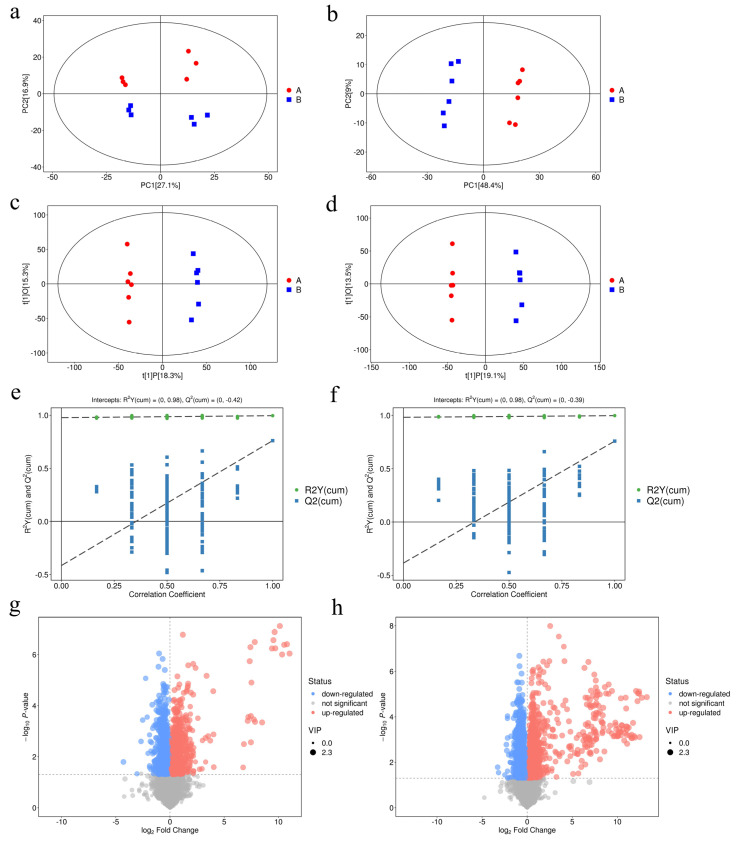
(**a**,**b**) Principal component analysis (PCA) under (**a**) positive ion mode (POS) and (**b**) negative ion mode (NEG). (**c**,**d**) orthogonal projections to latent structures-discriminate analysis (OPLS-DA) under (**c**) positive ion mode and (**d**) negative ion mode. (**e**,**f**) A permutation test was implemented to assess the robustness and predictive ability of the OPLS-DA model under (**e**) positive ion mode and (**f**) negative ion mode. Class (A) represents the control samples and class (B) represents BCS samples. (**g**,**h**) Volcano plots of metabolites under positive ion mode and negative ion mode, respectively (*n* = 6). The x-axis represents the Log2(fold change), and the y-axis represents the −Log10(*p*-value). The red points represent significantly upregulated metabolites, the blue points represent significantly downregulated metabolites, and the gray points represent metabolites showing no change.

Principal component analysis (PCA) was carried out in order to visualize the distribution and the grouping of the samples under both positive (Figure 4a) and negative (Figure 4b) ion modes. The 95% confidence interval in the PCA score plot was used as the threshold to identify any potential outliers in the dataset. Moreover, orthogonal projections to latent structures-discriminate analysis (OPLS-DA) were applied in order to visualize the group separation and to identify significantly changed metabolites (Figure 4c,d). A large separation was found between the control and the treatment groups, which is indicative of a significant classification effect. We further performed a permutation analysis in order to test the OPLS-DA model. The intercept between the Q2 regression line and the y-axis was less than 0 (−0.42 and −0.39, respectively), which indicated that the model was valid and reliable and there was no overfitting. Significant differences in the metabolite concentrations between the control and the BCS treatment groups were determined using OPLS-DA with Student’s *t*-tests. The metabolites with a VIP score > 1.228 and *p* < 0.05 (Student’s *t*-test) were considered to be differentially abundant (Supplementary Tables S2 and S3). Compared to the control group, 98 and 55 metabolites were significantly upregulated and 57 and 18 were significantly downregulated in the BCS treatment group under positive (Figure 4g) and negative (Figure 4h) ion modes, respectively.

### 3.5. Copper Depletion Induce Ferroptosis

**Figure 5:**
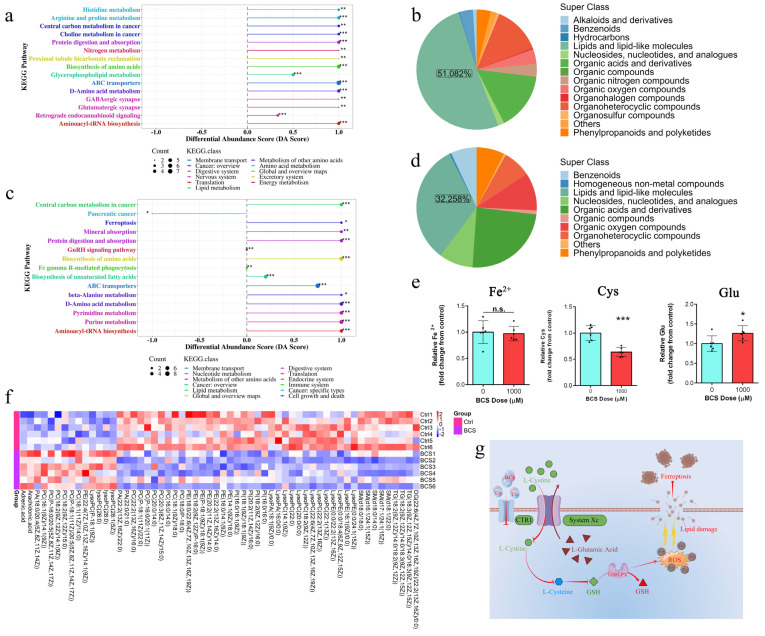
(**a**,**c**) Differential abundance score plot under the positive ion mode (**a**) and negative ion mode (**c**), respectively (*n* = 6). The x-axis represents the DA-Score, and the y-axis represents the KEGG metabolic pathway. The size of each dot represents the metabolite number, the dots are distributed on the right side of the central axis, and the longer the line segment is, the more the overall expression of the pathway tends to be up-regulated. * *p* < 0.05; ** *p* < 0.01; *** *p* < 0.001. (**b**,**d**) Differential metabolites were classified using the KEGG databases under positive ion mode (**b**) and negative ion mode (**d**), respectively (*n* = 6). (**e**) Measurement of Fe^2+^, cysteine, and glutamic contents in dermal papilla cells (DPCs) after 72 h of treatment with BCS (1000 µM) (*n* = 6/group). n.s. no significance; * *p* < 0.05; ** *p* < 0.01. (**f**) Clustering heat map of lipids and lipid-like molecules (*n* = 6). (**g**) Model depicting that the inhibited System Xc- reduces the uptake of cystine, driving ferroptosis.

The differential abundance analysis was generated in order to reflect the overall expression changes in all of the differentially abundant metabolites in each enriched pathway (Figure 5a,c). The differential metabolites were classified based on the KEGG (Kyoto encyclopedia of genes and genomes) database, in which 51.082% and 32.258% of the differential metabolites were classified as lipids and lipid-like molecules under positive (Figure 5b) and negative (Figure 5d) ion modes, respectively, among which, the ABC transporters, the biosynthesis of amino acids, the purine metabolism, and ferroptosis were the most significantly enriched pathways. However, the copper depletion had no significant effect on the intracellular content of Fe^2+^ (Figure 5e). The increased glutamate (Figure 5e) and the decreased cysteine (Figure 5e) contents indicate decreased glutathione synthesis. The loss of glycerophospholipids and sphingolipids, and the increased contents of AA, indicated the damage to the cell membrane that was caused by ferroptosis (Figure 5f). The model below shows that the inhibited System Xc- reduces the uptake of cystine, driving ferroptosis (Figure 5g). 

### 3.6. Copper Depletion Decreased the Sensitivity of DPCs to Erastin

**Figure 6:**
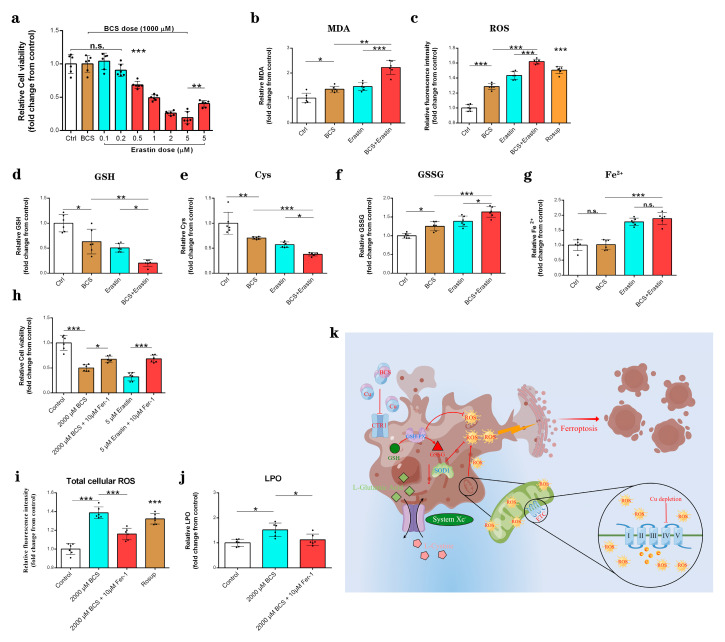
(**a**) A cell counting kit-8 (CCK-8) assay was used to assess the effect of the combination of BCS (1000 µM) and erastin (0.1~5 µM) on DPC viability after 48 h of treatment (*n* = 6/group). n.s. no significance; ** *p* < 0.01; *** *p* < 0.001. (**b**-**g**) Measurement of MDA, total of cellular ROS, GSH, cysteine, GSSG, and Fe^2+^, contents in dermal papilla cells (DPCs) after 48 h of treatment with BCS (1000 µM) and erastin (1 µM) (*n* = 6/group). n.s. no significance; * *p* < 0.05; ** *p* < 0.01; *** *p* < 0.001. (**h**) Effect of BCS (2000 µM) and the ferroptosis inhibitor Fer-1 (10 µM), the ferroptosis activator erastin (5 µM), and Fer-1 (10 µM) on cell death (*n* = 6/group). * *p* < 0.05; *** *p* < 0.001. (**i**-**j**) Measurement of total cellular ROS and LPO contents in DPCs after 48 h of treatment with BCS (2000 µM) and Fer-1 (10 µM) (*n* = 6/group). * *p* < 0.05; *** *p* < 0.001. (**k**) Model depicting the mechanism of ferroptosis induced by copper depletion.

The depletion of copper by BCS resulted in significantly reduced resistance of the DPCs to erastin compared to the control group (Figure 6a). Indeed, the combination of BCS and erastin significantly enhanced the erastin-induced ferroptosis events, including increased MDA levels (Figure 6b), increased total cellular ROS levels (Figure 6c), GSH depletion (Figure 6d), cysteine depletion (Figure 6e), and GSSG (Figure 6f) generation. However, it did not further increase the Fe2+ accumulation compared to the erastin-treated group (Figure 6g). The ferroptosis inhibitor ferrostatin-1 (Fer-1) partially prevented BCS-mediated cell death (Figure 6h) and inhibited the generation of the total cellular ROS (Figure 6i) and LPO (Figure 6j). The model below depicts the mechanism of ferroptosis that is induced by copper depletion (Figure 6h).

## 4. Discussion

Copper is a double-edged sword in that the excessive accumulation of free intracellular copper is harmful to cells, while a same effect is observed when intracellular copper contents are exhausted [9,29]. Excessive copper induces cell death by targeting lipoylated TCA cycle proteins [29]. However, the mechanism underlying how copper depletion leads to cell damage remains unclear. Here, we have shown that BCS-mediated copper depletion exhausts GSH and decreases the protein level of GPX4. In addition, our results have shown that BCS-mediated copper depletion significantly reduced the resistance of DPCs to erastin, while the ferroptosis inhibitor ferrostatin-1 (Fer-1) partially prevented BCS-mediated cell death. Together, BCS-mediated depletion of copper strongly enhances ferroptosis via mitochondrial perturbation and a reduction in antioxidative mechanisms.

In order to identify the highest BCS dose that could effectively deplete intracellular copper, we treated DPCs in vitro with multiple concentrations of BCS. Our results showed a dose-dependent reduction in copper levels with treatment (Figure 1a). Additionally, the BCS treatment did not affect the cell viability at dosages of up to 1000 µM (Figure 1b). We next determined the most appropriate time for BCS treatment and found that BCS treatment at 1000 µM consumed the intracellular copper concentrations after 24 h of treatment initiation (Figure 1c). These findings were consistent with those of Krishnamoorthy [30]. Copper uptake and transportation are strictly controlled by various cellular mechanisms. After copper uptake into cells through CTR1, several copper chaperone proteins deliver the copper to defined organelles and proteins (e.g., CCS transports copper to SOD1, while COX17 transports copper to complex IV) [28,29]. COX17 is a copper chaperone that showed similarity to CCS. Accordingly, the detected increase in COX17 mRNA levels was indicative for the depletion of mitochondrial copper (Figure 2a). Song et al. demonstrated that Sp1 functions as a copper sensor that regulates the CTR1 expression in response to variations in copper concentrations [31]. We believe that the increases in the mRNA expression levels of SP1 (Figure 1d) and CTR1 (Figure 1e) that were observed in this study likely occurred as a response to intracellular copper depletion. Moreover, western blot analysis further demonstrated that the protein expression of MTCO1 was reduced in BCS-treated DPCs (Figure 1f). Ramchandani [9] demonstrated that copper depletion did not affect the mRNA levels of either MTCO1 or COX4I1, which is consistent with our findings (Figure 2b,c). Although BCS-mediated copper depletion did not affect the protein level of cytochrome c (Figure 2d), it did lead to a significant decrease in the levels of cytochrome c oxidation (Figure 2e) and ATP production (Figure 2f). COX is the terminal enzyme of the mitochondrial ETC and is involved in the formation of the MMP, which is used by F_1_F_0_-ATPase to drive the production of ATP [32]. Our results showed that BCS-mediated copper depletion significantly decreased the MMP (Figure 3a), which is the main reason for the decrease in the ATP generation. As signaling molecules, ROS play an important role in cell proliferation and cell fate determination, but excessive ROS production can lead to irreversible cell damage and cell death [33,34]. While complex I and complex III are direct sites of ROS generation, perturbations of COX activity can indirectly increase the mitochondrial ROS production [35], which is consistent with our results (Figure 2h). In fact, elevated ROS levels induce oxidative stress and are, in combination with free Fe^2+^ and disturbed GPX4 activity, markers for ongoing ferroptosis [6,14,15].

Protein carbonylation is elevated in most cell types as a consequence oxidative stress [36]. Our results showed that BCS-mediated copper depletion significantly increased the content of protein carbonylation (Figure 3a). In addition, we detected an increase in the levels of LPO (lipid peroxide) and MDA (malondialdehyde) (Figure 3b,c). MDA and LPO levels are commonly known as a marker of lipid peroxidation, while lipid peroxidation is the main downstream feature of ferroptosis [37,38]. Cells maintain intracellular redox homeostasis through a complex, endogenous antioxidant defense system [15]. Given that copper is an essential co-factor for SOD1 [39], which is located in the cytosol and the intermembrane space of the mitochondria, we hypothesized that the BCS-mediated intracellular copper depletion led to a reduction in SOD1 activity (Figure 3d). Moreover, we detected the depletion of GSH (Figure 3e) and the inactivation of GSH-Px (Figure 3f). The transmission electron microscopy (TEM) showed that DPCs that were treated with BCS exhibited shrunken mitochondria and a reduced number of cristae (Figure 3g), which is a sign of malfunctioning mitochondria and excessive ROS production [6]. We speculated that the overproduction of ROS might lead to enhanced ferroptosis. Therefore, we continued to detect the protein levels of GPX4, which plays an essential role in ferroptosis, as it uses GSH to convert phospholipid hydroperoxides into lipid alcohols and inhibits the ROS-induced ferroptosis [40]. Yang reported that a large depletion of GSH can lead to the inhibition of GPX4 and the loss of endogenous cellular antioxidant capacity [41]. Accordingly, the western blot analysis confirmed a reduction in GPX4 (Figure 3h), although, Lee reported that AMPK activation can inhibit ferroptosis [6]. However, we observed an increase in AMPK phosphorylation at Thr172 (Figure 3h). We believe that copper depletion leads to mitochondrial dysfunction, which leads to ATP reduction and, finally, to AMPK activation.

A large number of studies have shown an intimate link between ferroptosis and metabolism [14,42]. We sought to determine the direct link between copper-depletion-mediated metabolic reprogramming and ferroptosis using LC–MS analysis. As expected, the copper depletion led to a significant change in the lipids and the lipid-like molecules (Figure 5c,d). We also detected an increase in the levels of the arachidonic acid (AA) and adrenic acid (AdA) (Figure 5h). In fact, AA and AdA are prone to be oxidized in order to generate lipid peroxide, thus representing the major inducers of ferroptosis [37]. Cysteine is the rate-limiting precursor of reduced glutathione, and the cystine/glutamate antiporter (System Xc-) undergoes a 1:1 transportation of L-glutamate for cystine [43]. Pitman [20] reported that glutamate levels affect the function of System Xc-, and higher contents of glutamate inhibit System Xc- transport [19,44]. Our data suggest that the accumulation of glutamate (Figure 5g) inhibited the function of System Xc-, which resulted in the increased intracellular depletion of cysteine (Figure 5g). Although cystine/glutamate can be interconverted intracellularly, the uptake of cystine from extracellular to intracellular is the dominant cause of the change in the cysteine content [45,46]. Cells have been shown to utilize additional carbon sources for energy supply, such as glutamine [47]. Metabolic reprogramming due to the decreased mitochondrial ATP production improves glutamine uptake and utilization. Our data suggest that the increased uptake of glutamine is the main reason for the increased content of glutamate. However, we found that copper depletion had no significant effect on the intracellular Fe^2+^ content (Figure 5g). Therefore, we suspect that the BCS-mediated depletion of copper enhances ferroptosis via a reduction in antioxidative capacities.

In order to further test our hypothesis, we used erastin (an inducer of ferroptosis) [41,48] and ferrostatin-1 (Fer-1) (the ferroptosis inhibitor) [49,50] to explore the mechanism of ferroptosis that was induced by copper depletion. As expected, erastin significantly induced cell death and ferroptosis events (Figure S1), which was consistent with previous research [41,48]. The depletion of copper by BCS resulted in significantly reduced resistance of DPCs to erastin compared to the control group and the erastin group (Figure 6a). The combined effects of BCS and erastin suggest that ferroptosis is involved in the BCS-induced reduction in cell viability. Indeed, the combined treatment with BCS and erastin showed a stronger effect on MDA (Figure 6b) and total cellular ROS (Figure 6c) production, GSH (Figure 6d) and cysteine (Figure 6e) depletion, and GSSG accumulation (Figure 6f), which are the hallmarks of ferroptotic cell death [51]. However, the combined interference with BCS and erastin showed no effect on Fe^2+^ accumulation (Figure 6g). Fer-1 is an active radical-trapping antioxidant and is widely used as an inhibitor of ferroptosis; moreover, the function of Fer-1 inhibits ferroptosis by inhibiting the lipid peroxidation [52,53]. As shown in Figure 6h, Fer-1 partially prevented BCS-induced cell death. In addition, Fer-1 significantly inhibited BCS-induced ferroptosis events, including the total cellular ROS (Figure 6i) production and the LPO (Figure 6g) generation, which again suggests the involvement of ferroptosis in BCS-induced cell death.

## 5. Conclusions

The present study has revealed an essential role for copper in intracellular redox homeostasis. Excessive ROS production, free Fe^2+^, and reduced GPX4 and SOD1 activity are crucial for the induction of ferroptosis. Copper depletion strongly enhanced ferroptotic cell death by disrobing important cellular antioxidative defense mechanisms and by excessive ROS production of the permutated mitochondria. In summary, this work identifies a direct link between ferroptosis and copper and contributes to an emerging paradigm of the involvement of transition metals in cell death.

## Data Availability

Data is contained within the article and supplementary material.

## Supplementary Materials

The following supporting information can be downloaded at: https://www.mdpi.com/article/10.3390/antiox11112084/s1. Table S1: Primer sequences of related genes; Table S2: Positively identified significantly differentially expressed metabolites in the dermal papilla cells after BCS treatment under the positive ion mode; Table S3: Positively identified significantly differentially expressed metabolites in the dermal papilla cells after BCS treatment under the negative ion mode; Figure S1: Erastin significantly induced ferroptosis.
