# Supplementary material for: Copper Depletion Strongly Enhances Ferroptosis via Mitochondrial Perturbation and Reduction in Antioxidative Mechanisms
Source: Antioxidants (Basel). 2022 Oct 22;11(11):2084. doi: 10.3390/antiox11112084 (PMC9687009; doi:10.3390/antiox11112084)
Supplement: Supplementary file 1 [file antioxidants-11-02084-s001.zip › Supplementary Figure. S1.pdf]

Supplementary Figure. S1: Erastin significantly induced ferroptosis.

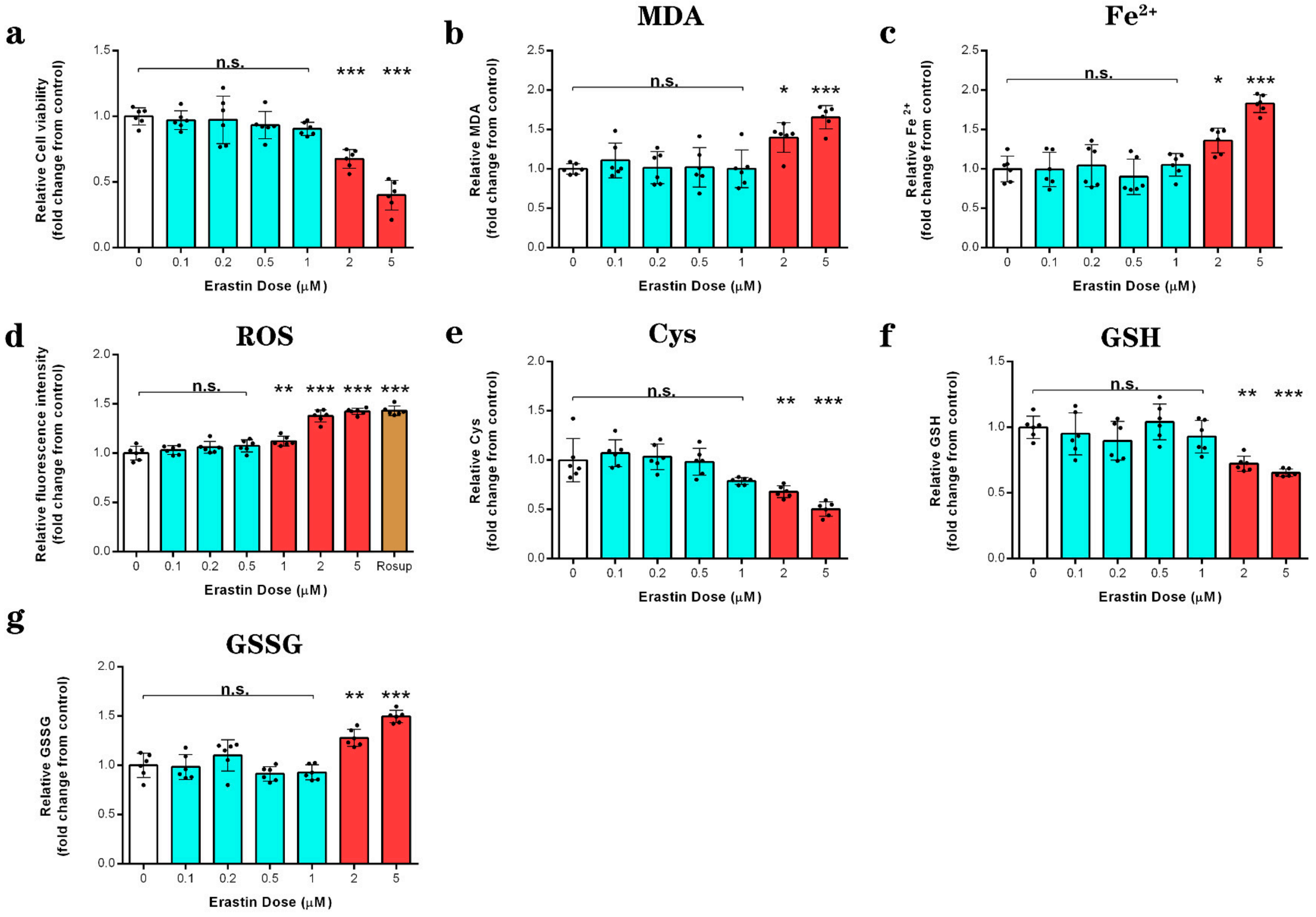

Supplementary Figure. S1. (a) A cell counting kit-8 (CCK-8) assay was used to assess the effect of different erastin (the ferroptosis activator) concentrations on DPC viability after 48 h of treatment (*n*=6/group). n.s. no significance; ***p<0.0001. **(b)** Measurement of MDA content in dermal papilla cells (DPCs) after 48 h of treatment with erastin (0~5 μM) (*n*=6/group). n.s. no significance; *$p < 0.05$; ***p<0.001. **(c)** Measurement of Fe^{2+} content in dermal papilla cells (DPCs) after 48 h of treatment with erastin (0~5 μM) (*n*=6/group). n.s. no significance; *$p < 0.05$; ***p<0.001. **(d)** Measurement of ROS content in dermal papilla cells (DPCs) after 48 h of treatment with erastin (0~5 μM) (*n*=6/group). n.s. no significance; **$p < 0.01$; ***p<0.001. **(e)** Measurement of cysteine content in dermal papilla cells (DPCs) after 48 h of treatment with

erastin (0~5 μM) (n=6/group). n.s. no significance; **$p < 0.01$; ***$p<0.001$. **(f)** Measurement of GSH content in dermal papilla cells (DPCs) after 48 h of treatment with erastin (0~5 μM) (n=6/group). n.s. no significance; **$p < 0.01$; ***$p<0.001$. **(g)** Measurement of GSSG content in dermal papilla cells (DPCs) after 48 h of treatment with erastin (0~5 μM) (n=6/group). n.s. no significance; **$p < 0.01$; ***$p<0.001$.
