# Supplementary material for: Copper Depletion Strongly Enhances Ferroptosis via Mitochondrial Perturbation and Reduction in Antioxidative Mechanisms
Source: Antioxidants (Basel). 2022 Oct 22;11(11):2084. doi: 10.3390/antiox11112084 (PMC9687009; doi:10.3390/antiox11112084)
Supplement: Supplementary file 1 [file antioxidants-11-02084-s001.zip › Supplementary material for blots.pdf]

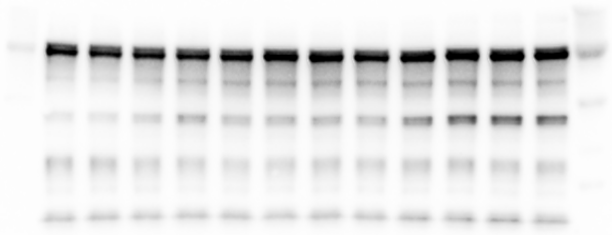

Figure S1. f. T-OXPHOS. Lane 1-3: BCS (1000, uM), Lane 4-6: BCS (500 uM), Lane 7-9: BCS (200 uM), Lane 10-12: Control.

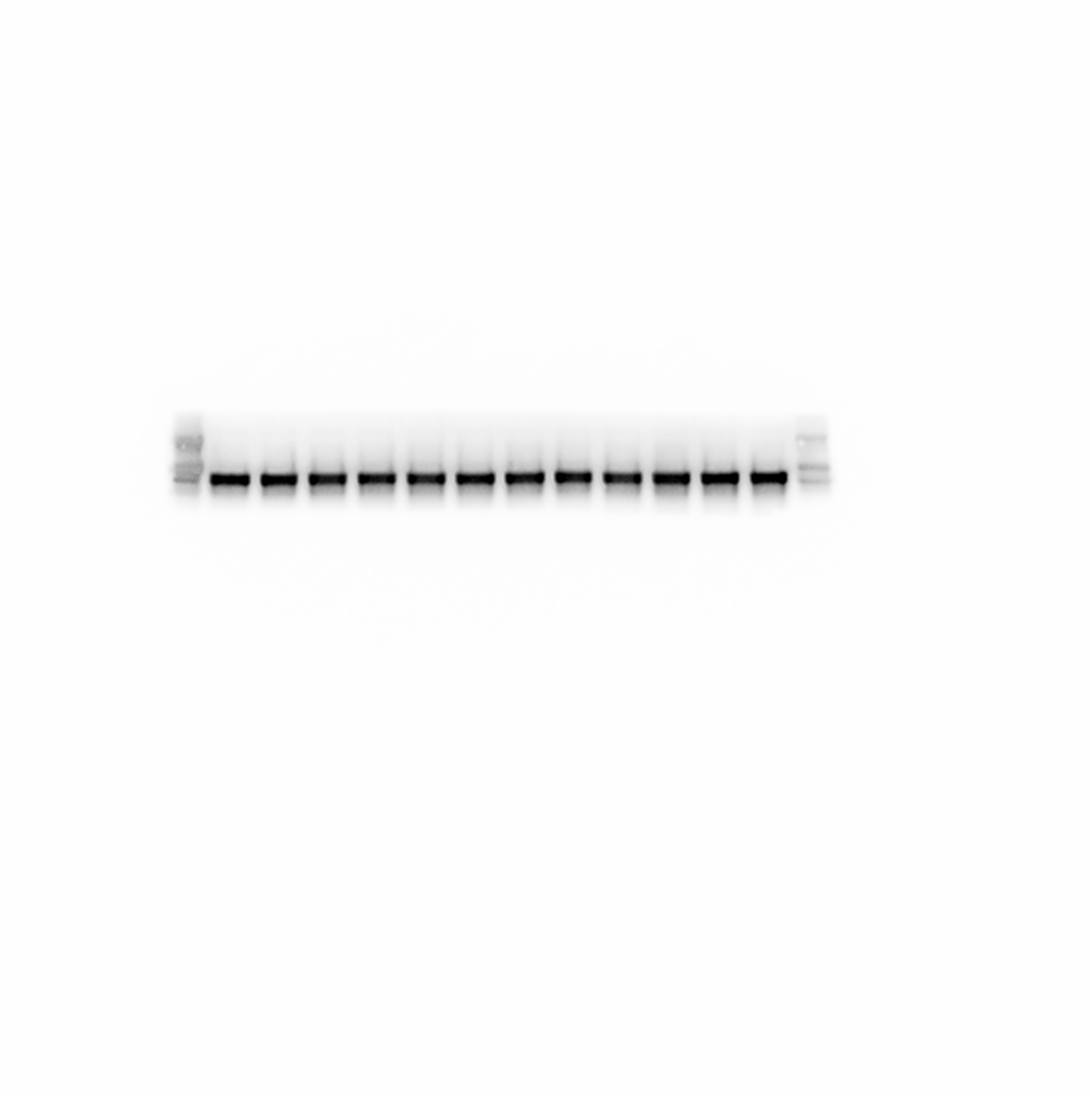

Figure S1f. GAPDH Lane 1-3: Control, Lane 4-6: BCS (200 uM), Lane 7-9: BCS (500 uM), Lane 10-12: BCS (1000, uM).

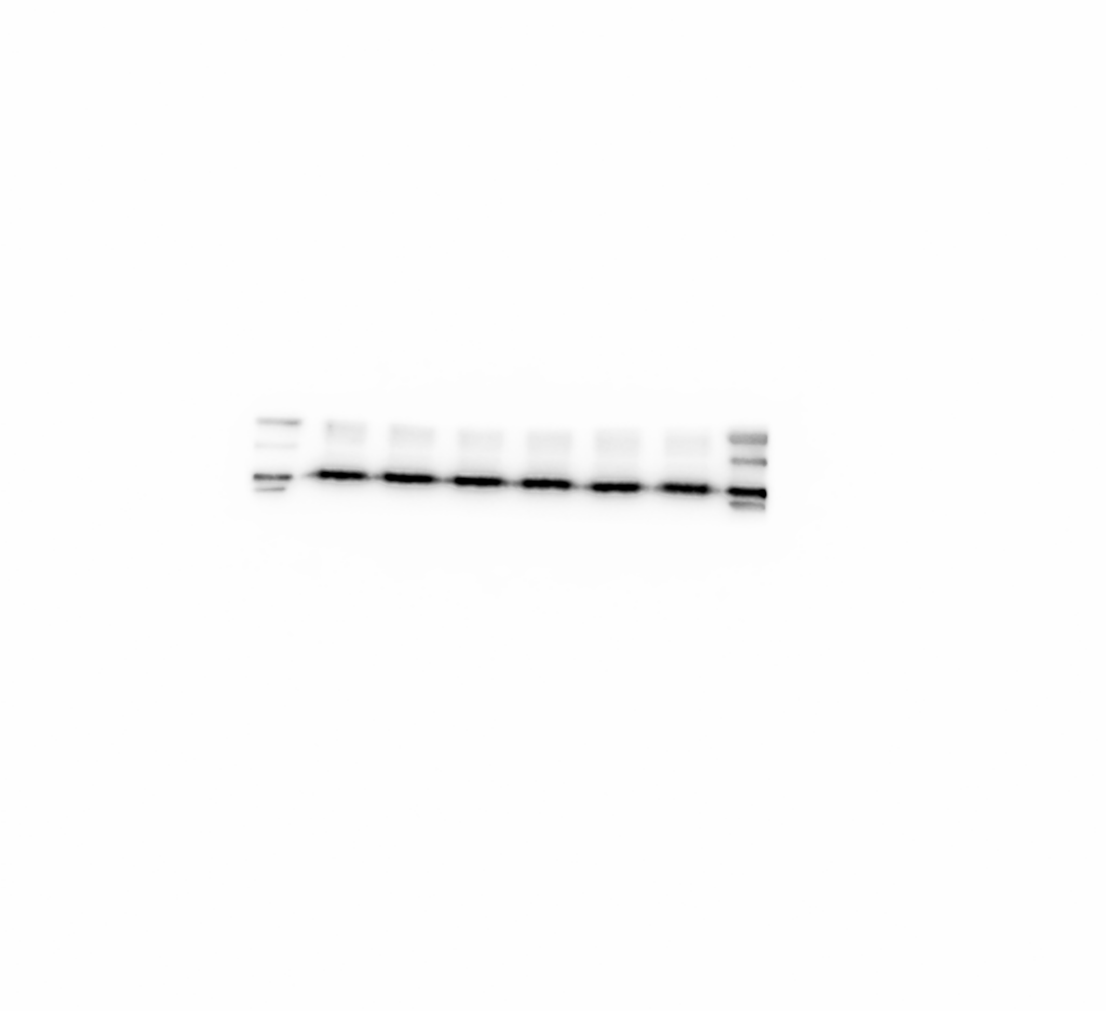

Figure S2e. Cytochrome c. Lane 1-3: Control, Lane 4-6: BCS (1000, uM).

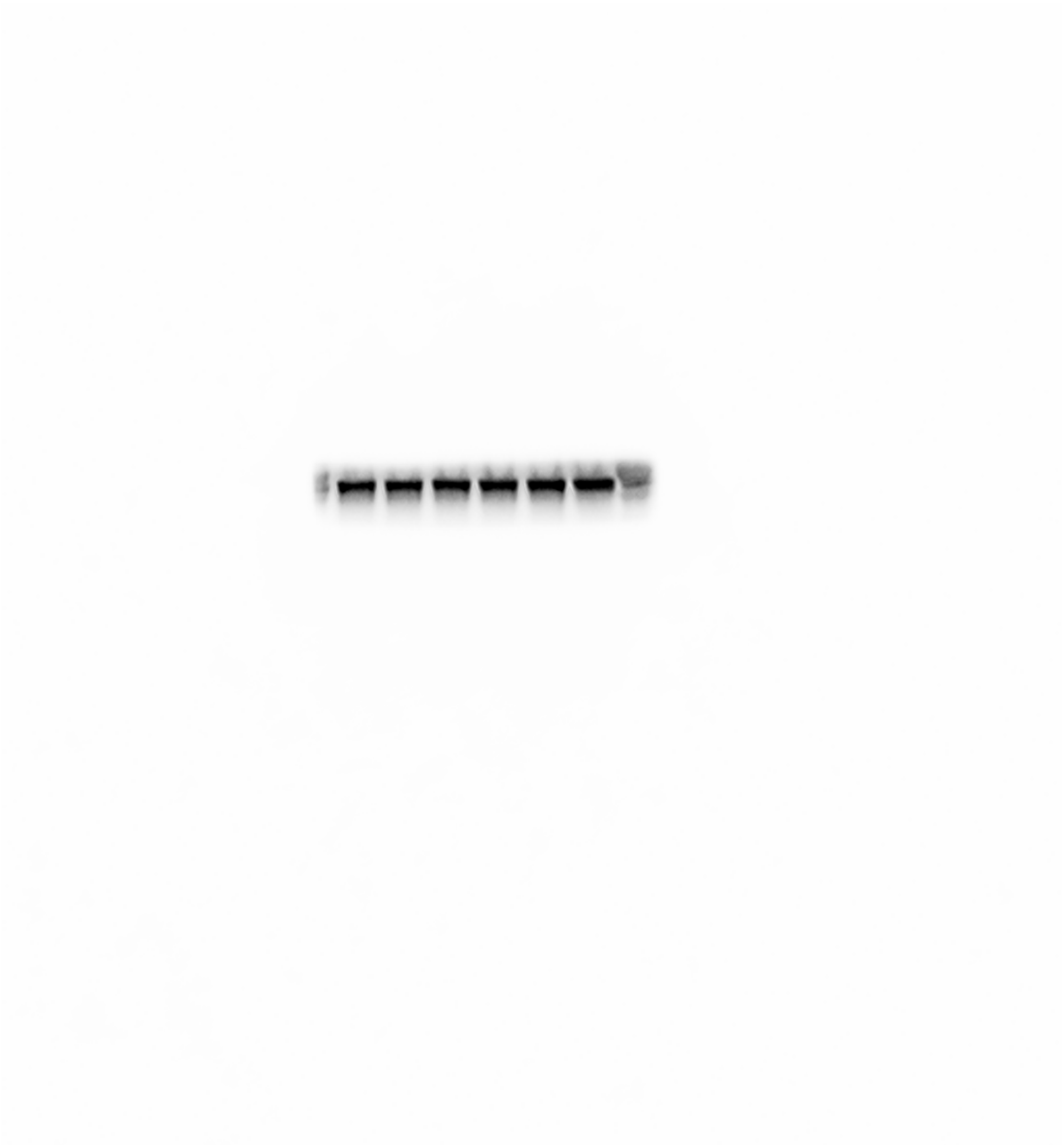

Figure S2e. GAPDH. Lane 1-3: Control, Lane 4-6: BCS (1000, uM).

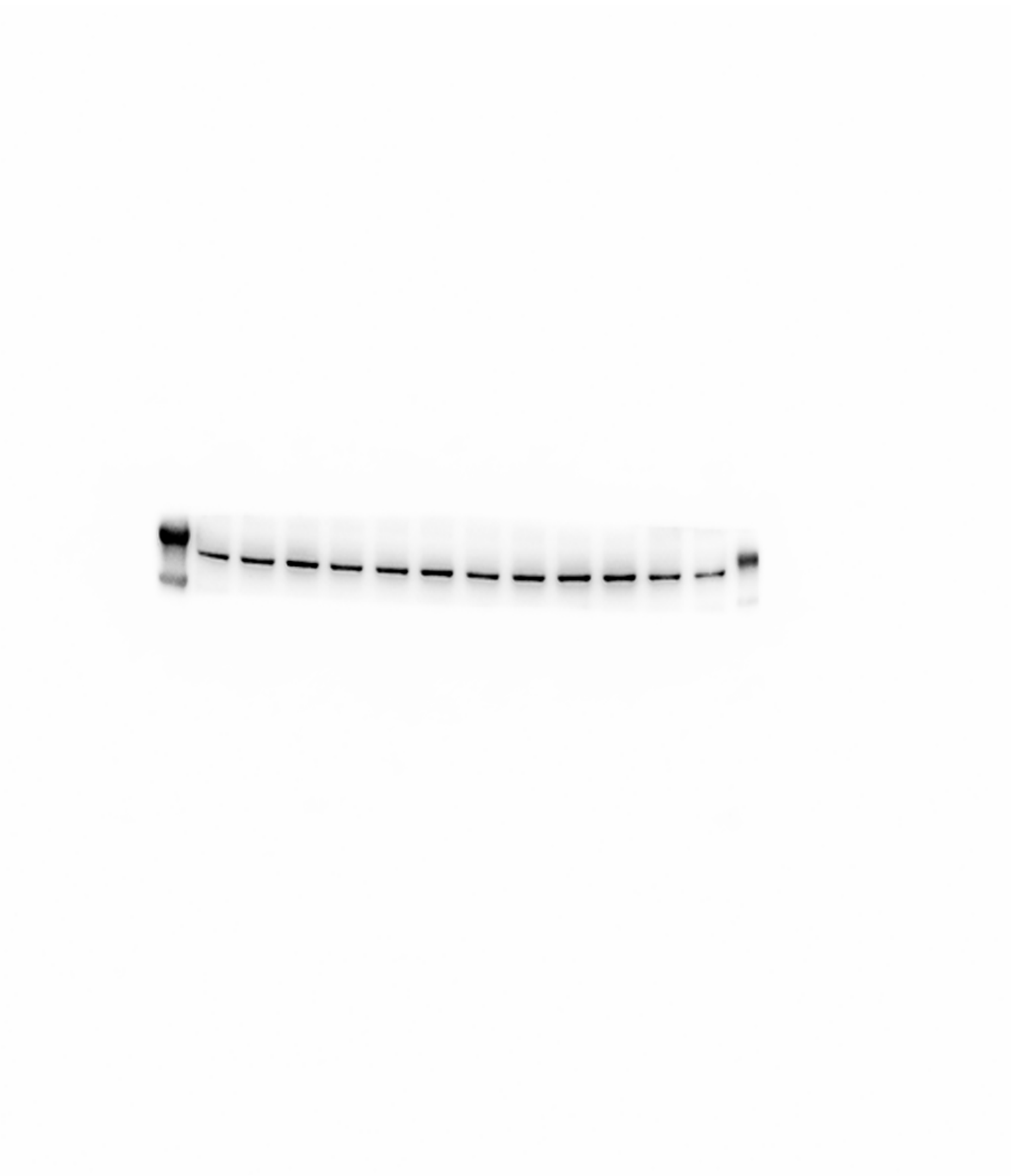

Figure S3h. AMPK. Lane 1-3: Control, Lane 4-6: BCS (1000, uM).

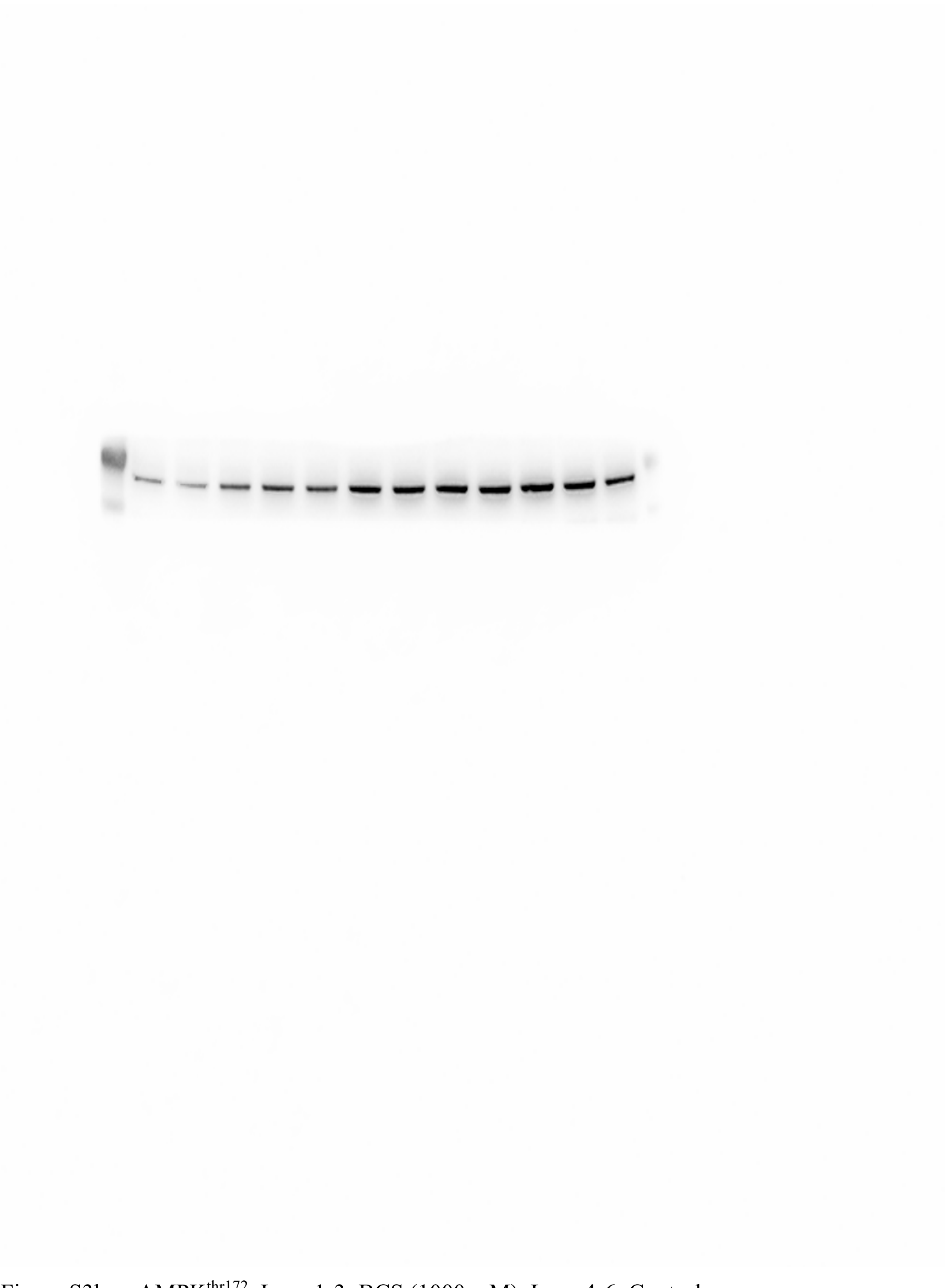

Figure S3h. p-AMPK^{thr172}. Lane 1-3: BCS (1000, uM). Lane 4-6: Control.

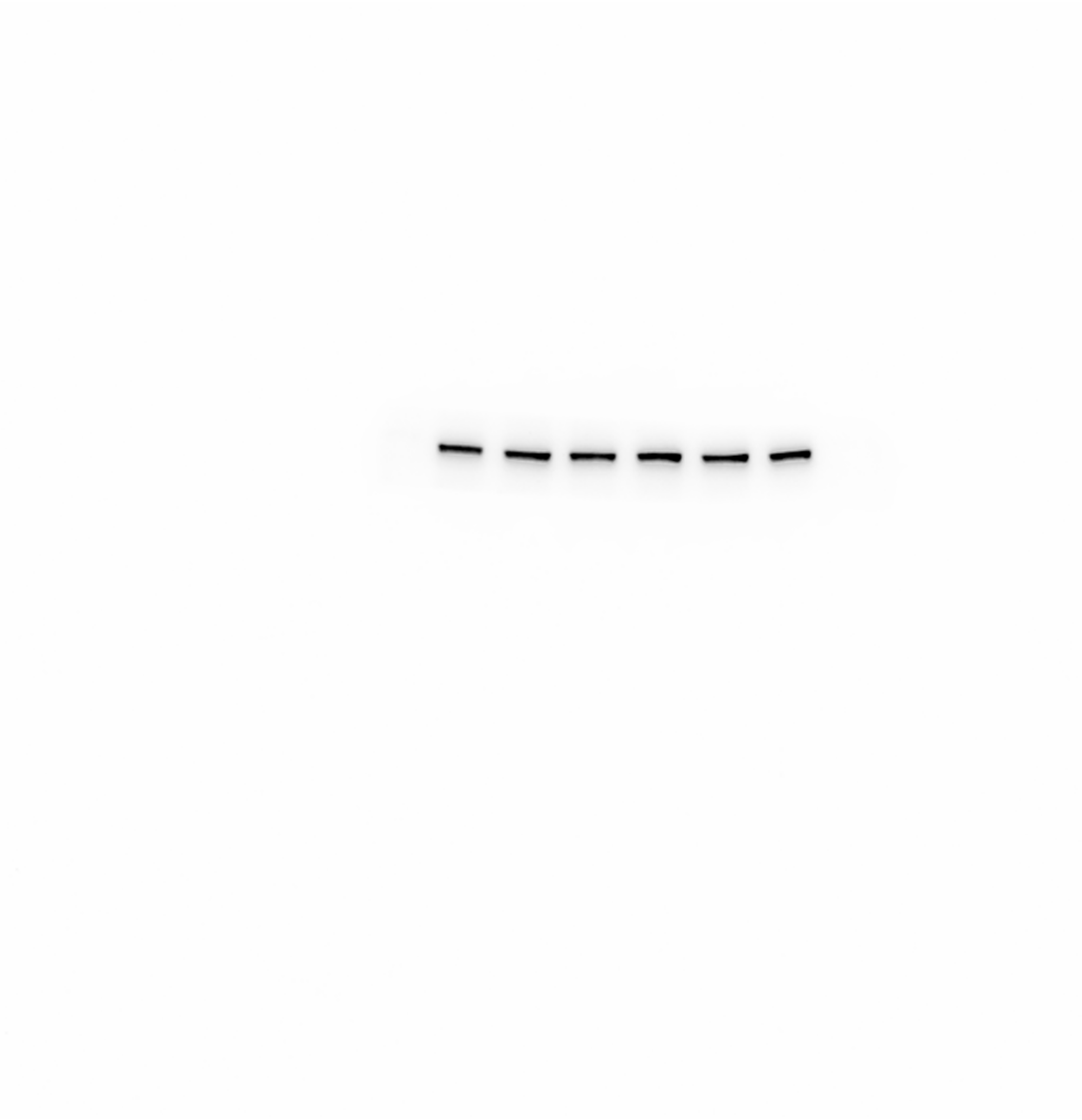

Figure S3h. GAPDH. Lane 1-3: Control. Lane 4-6: BCS (1000, uM).

.

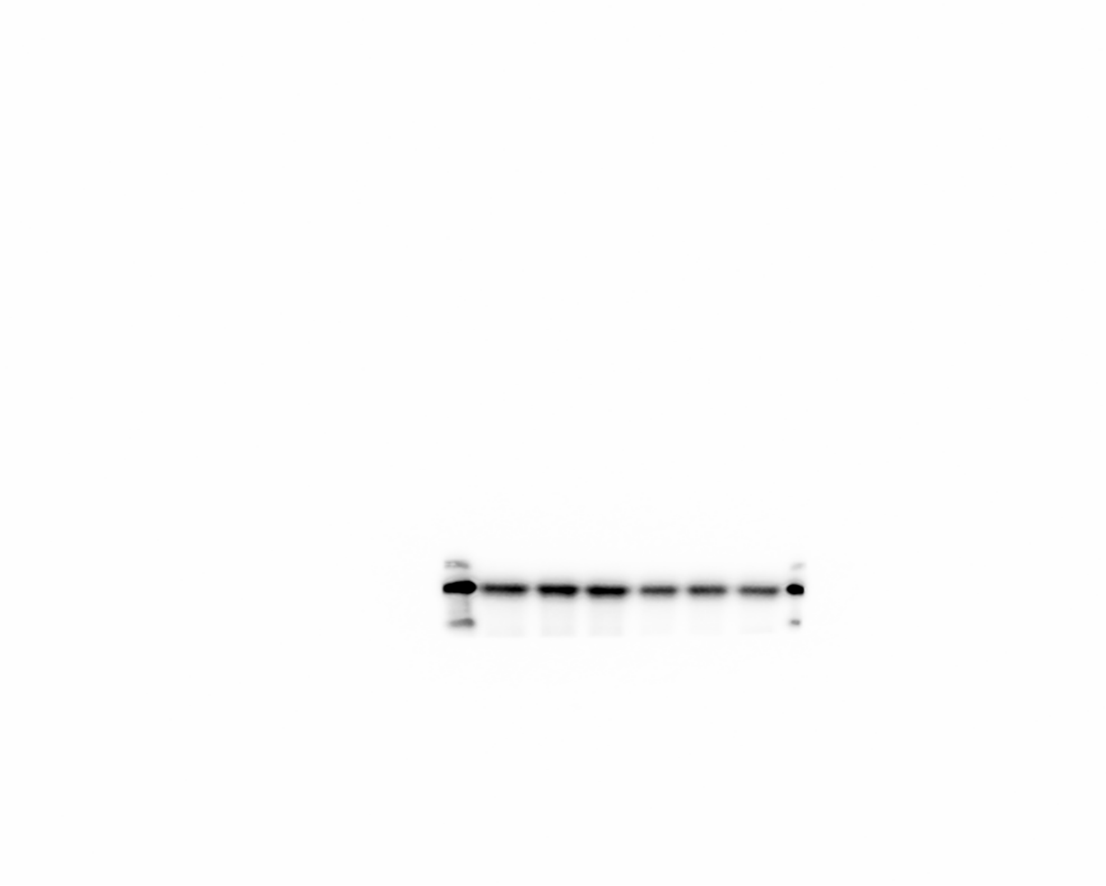

Figure S3h. GPX4. Lane 1-3: Control. Lane 4-6: BCS (1000, uM)
