# Supplementary material for: Copper Depletion Strongly Enhances Ferroptosis via Mitochondrial Perturbation and Reduction in Antioxidative Mechanisms
Source: Antioxidants (Basel). 2022 Oct 22;11(11):2084. doi: 10.3390/antiox11112084 (PMC9687009; doi:10.3390/antiox11112084)
Supplement: Supplementary file 1 [file antioxidants-11-02084-s001.zip › Supplementary Table S1.pdf]

Supplementary Table S1.

Primer sequences of related genes

Genes 1)	GenBank accession number	Primer sequences (5′ - 3′)	Product size/bp
MTCO1	NP_007551.1	F: 5′- CTATTTGGAGCTTGAGCTGGGATGG -3′<br>R:5′- AAGGCATGTGCGGTGACGATTAC -3′	128
COX17	XM_002716664.3	F: 5′- CAGGAGAAGAAGCCGCTGAAGC -3′<br>R:5′- GGGCCTCAATTAGATGTCCACAGTG -3′	141
COX4I1	NM_001361489.1	F: 5′- CCCTGTTCTTCATCGGCTTCACC -3′<br>R:5′- CACCTTCATGTCCAGCATCCTCTTG -3′	131
SP1	XM_002711128.3	F: 5′- CGAGGAAGTGGAGGCAACATCATC -3′<br>R:5′- TGGTGGTAGTCGTCGTGGAGTAG -3′	352
CTR1	XM_017345238.1	F: 5′- TGACCACCACAGCCTCACACTC -3′<br>R:5′- GCTGATGACCACCTGGATGATATGC -3′	368
GAPDH	NM_001082253.1	F: 5′- CACCAGGGCTGCTTTTAACTCT -3′<br>R:5′- CTTCCCGTTCTCAGCCTTGACC -3′	163
β-actin	XM_002722894.3	F: 5′- CGCAGAAACGAGACGAGATT -3′<br>R:5′- GCAGAACTTTGGGGACTTTG -3′	123

[1] MTCO1 = cytochrome c oxidase subunit 1; COX17 = cytochrome c oxidase copper chaperone; COX4I1 = cytochrome c oxidase subunit IV; SP1 = Sp1 transcription factor; CTR1 = copper transporter 1; GAPDH = glyceraldehyde phosphate dehydrogenase; β-actin = actin alpha.
