# Supplementary material for: Copper Depletion Strongly Enhances Ferroptosis via Mitochondrial Perturbation and Reduction in Antioxidative Mechanisms
Source: Antioxidants (Basel). 2022 Oct 22;11(11):2084. doi: 10.3390/antiox11112084 (PMC9687009; doi:10.3390/antiox11112084)
Supplement: Supplementary file 1 [file antioxidants-11-02084-s001.zip › Supplementary Table S2.pdf]

Supplementary Table.S2

Positively identified significantly differentially expressed metabolites in the dermal papilla cells after BCS treatment under postive ion mode (n = 6).

Metabolite	rt	mz	VIP	P-VALUE	FC	LOG_FC	Regulate
Adenine	169.8	136.06	1.44008	0.039020972	1.44874	0.534798304	up
Hypoxanthine	178.6	137.05	1.80995	0.006727798	1.17106	0.227821044	up
Homocysteine thiolactone	183.4	118.03	1.59326	0.010014499	1.87283	0.905221406	up
L-Carnitine	377.7	162.11	1.73944	0.005278174	1.29552	0.37352674	up
Beta-Aminopropionitrile	448.5	71.06	1.45449	0.030760466	1.15006	0.20170906	up
Celereoin	178.6	263.1	2.15981	1.24449E-05	182.676	7.513140176	up
Propionylcarnitine	302.4	218.14	1.89221	0.001293021	1.49243	0.577658459	up
Zymonic acid	179.1	159.03	1.8304	0.002435857	1.27128	0.346287282	up
O-Acetylcarnitine	328.6	204.12	1.67524	0.004621277	1.41582	0.501637761	up
Choline	319.1	104.11	2.10659	0.000138227	1.29177	0.369344688	up
Falcarinone	399.3	241.15	1.3547	0.048676173	1.1889	0.249623218	up
Imidazole-4-acetaldehyde	304.6	111.06	1.69457	0.006226854	1.13342	0.180679705	up
Thiamine	437.4	265.11	1.9719	0.000478724	1.86827	0.90170069	up
2-Methyl-3-(2-propenyl)pyrazine	237.9	135.09	1.99063	0.000349984	1.24017	0.310536992	up
5-Acetyl-2,4-dimethyloxazole	434	140.07	1.87326	0.001967334	0.63383	-0.65782702	down
Glycyl-Valine	298	175.11	1.92074	0.001619605	1.53991	0.622849679	up
LysoPE(0:0/18:4(6Z,9Z,12Z,15Z))	226	474.26	1.62298	0.014505674	0.8222	-0.282443532	down
Neopellitorine A	436	230.15	1.53203	0.025285014	0.81941	-0.287349405	down
Phenylalanyl-Valine	190.9	265.16	1.50473	0.045095101	1.33099	0.412495131	up
Graveoline	412.3	280.09	1.7817	0.002814353	0.63367	-0.658204289	down
Isoleucyl-Isoleucine	198	245.19	1.79414	0.008620097	1.48879	0.574141857	up
8-Hydroxy-7-methylguanine	206.2	182.07	1.86983	0.000728317	1.56784	0.648782325	up

Acadesine	293.1	259.1	1.31398	0.047225857	1.26286	0.336690844	up
Leucyl-Threonine	248.2	233.15	1.67768	0.013537691	1.6019	0.679782086	up
4-Guanidinobutanoic acid	382	146.09	1.60962	0.016250195	1.16388	0.218945338	up
Methyl 3,4-dicaffeoylquinate	37.33	545.18	1.54746	0.000279799	0.23273	-2.103274357	down
Prolyl-Threonine	343.3	217.12	1.97689	0.000487996	1.42112	0.50702784	up
Phenylalanyl-Serine	247.4	253.12	1.71422	0.009256056	1.47775	0.563401776	up
L-Glutamic acid	420.1	148.06	1.44744	0.041208102	1.26179	0.335466176	up
PI(20:2(11Z,14Z)/16:0)	206.2	863.57	2.10617	9.23363E-05	0.75353	-0.40825445	down
L-Proline	330.2	116.07	1.40678	0.026200261	1.26005	0.333480763	up
2-Galloylglucose	225.8	333.08	1.62201	0.013351112	1.24899	0.320767322	up
H-THR-PHE-OH	220.9	267.13	1.81512	0.005862299	1.48219	0.567728088	up
Pyridoxine	107.3	170.08	1.4767	0.031875862	1.33771	0.419763092	up
PI(18:2(9Z,12Z)/16:0)	207.5	835.54	1.58909	0.022182025	0.84238	-0.247458834	down
2-Amino-3,8-dimethyl-3H-imidazo[4,5-f]quinoxaline	365.2	214.11	1.92394	0.001453664	1.87708	0.908489289	up
1,6-Dimethoxy-9H-carbazole-3-carboxaldehyde	302.3	256.09	1.26022	0.035728747	0.80442	-0.313973071	down
PC(18:2(9Z,12Z)/14:1(9Z))	172	728.52	1.28037	0.038908243	1.28589	0.362768327	up
N-Acetyl-2,6-diethylaniline	217.6	192.14	1.58041	0.014331922	0.90801	-0.139223772	down
4-Hydroxy-1H-indole-3-acetonitrile	57.81	173.08	1.76201	0.007232912	1.52509	0.608898523	up
LysoPE(16:1(9Z)/0:0)	225.2	452.28	1.56717	0.02026487	0.79127	-0.337750315	down
LysoPC(P-18:1(9Z))	207.5	506.36	2.03413	0.000144264	1.28039	0.356587818	up
Schleicherastatin 3	24.14	447.38	1.51899	0.000940658	4.25488	2.089119267	up

LysoPE(0:0/24:1(15Z))	214.3	564.4	1.51159	0.022319934	0.86286	-0.212808395	down
Indole-5,6-quinone	351.5	148.04	1.839	0.000932361	1.51989	0.603968046	up
Prolyl-Alanine	350.6	187.11	2.05963	0.000191876	1.52338	0.60727565	up
Trans-2, 3, 4-Trimethoxycinnamate	56.74	239.09	2.14506	0.002344609	13.1208	3.713782496	up
Isodesmosine	218.8	526.29	1.64752	0.012447659	0.83651	-0.257544827	down
Isoleucyl-Valine	209.2	231.17	1.84409	0.005456248	1.41965	0.505535508	up
Valyl-Tryptophan	467.5	304.16	1.48579	0.036094357	1.38785	0.472855486	up
PC(20:0/14:0)	182.5	762.6	1.40776	0.037212882	0.85767	-0.22151295	down
Valyl-Arginine	388.3	274.19	1.70877	0.010796345	1.66812	0.738222725	up
Koeniginequinone A	417.7	242.08	1.43094	0.019607647	0.84589	-0.241452213	down
N-Ornithyl-L-taurine	363	240.1	1.92818	0.012104684	2.21126	1.144869485	up
(-)-Dioxibrassinin	348.4	269.04	1.89132	0.001182039	1.42279	0.508717725	up
5-(3',4'-dihydroxyphenyl)-gamma-valerolactone-3'-O-glucuronide	228.7	399.13	2.12969	5.95485E-05	1.90315	0.928387093	up
Phenylalanyl-Methionine	184.9	297.13	1.43046	0.029821379	3.58954	1.843797266	up
O-Acetyl-L-serine	406.6	148.06	1.43748	0.043340976	1.34982	0.432763097	up
L-Palmitoylcarnitine	199.3	400.34	1.93051	0.001050968	1.50731	0.591978193	up
Prolylphenylalanine	251.9	263.14	2.01474	0.000485302	2.03471	1.02482024	up
6-Hydroxy-1H-indole-3-acetamide	274.3	191.09	1.84066	0.003801168	1.25249	0.324798283	up
N-Succinyl-2-amino-6-ketopimelate	247.4	290.09	1.46612	0.026152539	1.23678	0.30658315	up
LysoPC(14:1(9Z))	223.6	466.29	1.37131	0.04526968	0.82248	-0.281941385	down

Elaidic carnitine	196.7	426.36	1.33017	0.042268665	1.24324	0.31410092	up
Glycylalanylprolylmethionylphenylalanylvalinamide	260.2	620.33	1.58622	0.011467449	0.74534	-0.424032764	down
2-(4-Methyl-5-thiazolyl)ethyl hexanoate	381.9	242.12	1.59047	0.01113629	1.37394	0.458316313	up
N-Carbamoyl-2-amino-2-(4-hydroxyphenyl) acetic acid	316.9	211.07	1.86578	0.001593623	1.86664	0.900442583	up
Glutaminyl-Gamma-glutamate	376.1	275.14	1.94384	0.002692577	1.61215	0.688986236	up
PC(20:3(8Z,11Z,14Z)/15:0)	171.3	770.57	1.49963	0.030684999	0.71145	-0.491166222	down
5-Acetyl-2,4-dimethylthiazole	134.3	156.05	1.42501	0.023624884	1.62864	0.703670745	up
3-(4-Methyl-3-pentenyl)thiophene	125.4	167.09	1.80062	0.000310245	5.95158	2.573272271	up
PC(16:0/14:0)	105.4	706.54	1.45424	0.031761369	0.41788	-1.258831801	down
3,4-Dihydroxyphenyllactic acid methyl ester	183.2	213.07	1.48197	0.017383282	2.34865	1.231830539	up
Gamma-glutamyl-L-putrescine	471.9	218.15	1.67161	0.015675367	1.3614	0.44509136	up
Carnosine	446.4	227.11	1.71655	0.004136437	1.38892	0.473958633	up
3-beta-Hydroxy-4-beta-methyl-5-alpha-cholest-7-ene-4-alpha-carbaldehyde	195.8	429.38	1.36907	0.043712263	1.41485	0.500647467	up
o-Tyrosine	503.9	182.08	1.76827	0.012535205	1.58499	0.664476329	up
Phenylalanyl-Lysine	357.1	294.18	1.53469	0.041500647	1.48329	0.56880522	up
PC(P-18:1(11Z)/20:5(	55.99	790.58	1.42765	0.022795158	1.72765	0.788807947	up

5Z,8Z,11Z,14Z,17Z))							
Propyl 2-furoate	223.3	155.07	1.8149	0.003248711	1.34392	0.426444126	up
LysoPC(22:0)	209.5	580.43	1.93933	0.001059637	0.73876	-0.436828995	down
1-Oleoylglycerophosphoinositol	253	599.32	1.59083	0.016283979	0.78907	-0.341783049	down
PE(18:0/22:6(4Z,7Z,10Z,13Z,16Z,19Z))	168.2	792.55	1.47378	0.039706088	0.6985	-0.517668433	down
PC(18:2(9Z,12Z)/15:0)	34.86	744.55	1.41099	0.038748901	1.18579	0.245851126	up
Dethiobiotin	302.4	215.14	1.84111	0.001423041	1.47338	0.559129063	up
Eriojaposide B	248.8	517.26	1.53978	0.049133578	0.67631	-0.564246135	down
PC(P-16:0/20:1(11Z))	66.66	772.62	1.63299	0.015386782	0.77031	-0.376489678	down
H-PHE-PRO-OH	199.6	263.14	2.05832	0.000200455	1.84168	0.881022094	up
SM(d18:0/18:0)	198.5	733.62	1.71397	0.007026946	0.68249	-0.551110553	down
Glucosyl (2E,6E,10x)-10,11-dihydroxy-2,6-farnesadienoate	314.1	433.25	1.45051	0.041157648	1.31658	0.396789946	up
PE(18:2(9Z,12Z)/P-16:0)	170.2	700.53	1.49674	0.031608623	0.65314	-0.614527039	down
LysoPC(20:0/0:0)	212.2	552.4	1.52036	0.020027272	0.82414	-0.279034012	down
CPA(18:2(9Z,12Z)/0:0)	361.6	417.25	2.15546	1.46119E-05	1.82416	0.867231378	up
PC(P-18:1(11Z)/22:0)	197.8	828.7	1.41914	0.046692034	0.62211	-0.68475103	down
PC(16:1(9Z)/14:1(9Z))	174.2	702.51	1.71278	0.010583391	1.45504	0.541059165	up
L-Glutamine	420.2	147.08	1.73356	0.007456179	1.68283	0.750892058	up
PC(22:2(13Z,16Z)/16:0)	166.3	814.63	1.7098	0.007401427	0.79492	-0.331120372	down
Lanthionine ketimine	245	190.01	1.69632	0.007268295	1.24321	0.314069948	up
LysoPC(18:2(9Z,12Z))	216.5	520.34	1.61274	0.014803211	0.85784	-0.221218315	down
Cytidine	256.1	244.09	1.46885	0.020099624	1.15984	0.213930096	up
Garciduol A	367.5	487.1	1.60857	0.011572185	3.37533	1.755026726	up

D-1-Amino-2-pyrrolidinecarboxylic acid	328.6	131.08	1.90943	0.001034179	1.22932	0.297856252	up
Alanyl-Gamma-glutamate	350.6	218.11	2.03015	0.000399917	1.66122	0.732247005	up
Theasapogenol A	207.6	507.36	1.98513	0.000380794	1.28255	0.359014273	up
PC(P-16:0/20:5(5Z,8Z,11Z,14Z,17Z))	106.9	764.56	1.43415	0.019088388	1.61259	0.689383903	up
lysoPC(28:0)	158.3	664.53	1.75141	0.005328831	1.70027	0.765764232	up
SM(d16:1/24:1(15Z))	199.3	785.66	1.94012	0.000862204	0.62478	-0.67857085	down
PE(P-18:1(9Z)/18:1(9Z))	167.4	728.56	1.69116	0.013115052	0.61605	-0.698872609	down
Auraptenol	349.8	243.1	1.61352	0.013402619	1.21502	0.280979281	up
SM(d18:1/22:0)	199.5	787.67	1.48639	0.037036374	0.59078	-0.75929703	down
8-Hydroxyguanine	249.1	168.05	1.69946	0.007834911	1.30714	0.386418436	up
PC(18:1(9Z)/18:0)	168.2	788.61	1.60021	0.013443121	0.75694	-0.401739786	down
PC(18:1(11Z)/14:0)	193.7	732.56	1.43918	0.033759239	1.246	0.31730256	up
PE(22:2(13Z,16Z)/14:0)	170.6	744.55	1.63417	0.012033184	0.7524	-0.41043599	down
SM(d18:0/14:0)	201.3	677.56	1.67387	0.006956693	0.86183	-0.214533051	down
Beta-Tyrosine	368.9	182.08	1.73286	0.007946417	1.3071	0.386374245	up
(E)-1-Cinnamoylpyrrolidine	337.4	202.12	1.40576	0.005080458	2.10628	1.074697972	up
SM(d17:1/24:1(15Z))	199	799.67	1.68636	0.00954313	0.65925	-0.601103842	down
Cohibin C	208.1	577.52	1.9942	0.000546536	0.7553	-0.404878266	down
LysoPC(22:6(4Z,7Z,10Z,13Z,16Z,19Z))	213.2	568.34	1.54564	0.019089482	0.87795	-0.187791964	down
PE(20:2(11Z,14Z)/14:0)	173.7	716.52	2.1702	1.84115E-05	0.82881	-0.27088822	down
lysoPC(26:0)	178.1	636.5	1.88196	0.003138278	1.42242	0.508350481	up
lysoPC(28:1(5Z))	175.3	662.51	2.14789	0.000874988	1.83981	0.879553713	up

LysoPC(22:2(13Z,16Z))	210.4	576.4	1.94136	0.001153228	0.69792	-0.518875224	down
PE(14:1(9Z)/18:0)	175.6	690.51	2.20955	3.98876E-06	0.71899	-0.475962291	down
PE(22:4(7Z,10Z,13Z,16Z)/14:1(9Z))	134.1	738.51	1.42237	0.044941646	1.97043	0.97851397	up
L-Lysine	575.7	147.11	1.97844	0.000725578	1.37969	0.464342661	up
LysoPC(22:1(13Z))	209.5	578.42	2.17238	3.5608E-05	0.66	-0.599453119	down
PI(18:1(9Z)/18:1(9Z))	205.6	880.59	2.08919	8.57604E-05	0.7774	-0.363279292	down
Ginsenoyne E	278.6	259.17	1.51558	0.036621485	1.75622	0.81247115	up
LysoPE(0:0/22:2(13Z,16Z))	215	534.36	1.55691	0.016845189	0.80441	-0.314003061	down
PA(22:2(13Z,16Z)/22:0)	165.5	813.62	1.7758	0.003347722	0.87364	-0.194891349	down
Glucosylceramide (d18:1/16:0)	48.34	700.57	1.87694	0.006100386	0.64639	-0.629521069	down
2-Methyl-1-methylthio-2-butene	330.1	117.07	1.38075	0.031607899	1.27092	0.345874918	up
PA(22:0/21:0)	208.2	803.66	2.18457	1.94143E-05	0.77356	-0.370408175	down
Phenylalanyl-Alanine	219	237.12	1.35854	0.005261705	3.58633	1.84250731	up
Ganoderic acid S	300.6	513.37	1.47554	0.014356511	1.42089	0.506790549	up
TG(18:2(9Z,12Z)/14:0/18:3(9Z,12Z,15Z))	197.3	825.68	1.7223	0.007728729	0.63755	-0.64939621	down
TG(18:3(9Z,12Z,15Z)/14:0/18:3(9Z,12Z,15Z))	197.3	823.67	1.88431	0.001955157	0.68473	-0.546385115	down
PC(18:0/P-16:0)	324.9	746.61	1.41296	0.046030816	0.68241	-0.551283539	down
PA(18:0/20:4(5Z,8Z,11Z,14Z))	205.2	742.53	1.50684	0.019780501	1.53017	0.613688883	up
DG(22:6(4Z,7Z,10Z,13Z,16Z,19Z)/22:2(13Z,16Z)/0:0)	145	721.6	1.43656	0.022520718	0.50091	-0.9973649	down
Nigellicine	295.4	247.11	2.17075	3.14737E-05	2.41962	1.274779436	up
PE(16:0/14:1(9Z))	178.4	662.48	1.38314	0.016012113	0.53475	-0.90305586	down

Methyl helianthenoate F glucoside	256.2	355.14	1.56536	0.017876284	1.29422	0.372078161	up
2-(4-Methyl-5-thiazolyl)ethyl propionate	251.3	200.08	1.61881	0.01497581	1.39421	0.479452702	up
PI(16:0/16:1(9Z))	209.5	809.52	1.42064	0.034507847	0.82355	-0.280076193	down
2,3-Dihydro-5-(5-methyl-2-furanyl)-1H-pyrrolizine	372.5	188.1	2.09659	7.34171E-05	1.47892	0.56453972	up
Physalolactone	248.7	539.24	1.78494	0.013533539	0.56443	-0.825138582	down
Eujambin	373.2	659.11	1.73093	0.004780367	1.50115	0.586066753	up
CerP(d18:1/26:0)	200.4	758.63	1.98983	0.000322979	0.68182	-0.55254576	down
5-Ethoxysorgoleone 358	351.3	373.24	1.62256	0.013073051	1.26928	0.34400899	up
N-Acetoxymethylflindersine	338.1	300.12	1.69239	0.010567769	0.75586	-0.403805015	down
Decarbamoylsaxitoxin	129.6	257.14	1.39513	0.036005429	1.48475	0.570223203	up
TG(18:2(9Z,12Z)/14:0/18:2(9Z,12Z))	197.3	827.7	1.46042	0.043464744	0.62194	-0.685157868	down

RT: Retention time; M/Z: mass charge ratio; VIP: Variable Importance for Projection, one indicator reflecting the capability of the variables to explain Y, FC: Fold change; LOG_FC: LOG_Fold change.
