# Supplementary material for: Copper Depletion Strongly Enhances Ferroptosis via Mitochondrial Perturbation and Reduction in Antioxidative Mechanisms
Source: Antioxidants (Basel). 2022 Oct 22;11(11):2084. doi: 10.3390/antiox11112084 (PMC9687009; doi:10.3390/antiox11112084)
Supplement: Supplementary file 1 [file antioxidants-11-02084-s001.zip › Supplementary Table S3.pdf]

Supplementary Table.S3

Positively identified significantly differentially expressed metabolites in the dermal papilla cells after BCS treatment under negative ion mode (n = 6).

Metabolite	rt	mz	VIP	P-VALUE	FC	LOG_FC	Regulate
Erucic acid	35.942	337.31	1.41485	0.038991979	0.87308	-0.19581964	down
Eicosadienoic acid	36.522	307.26	1.47148	0.029850371	0.89701	-0.15679814	down
Adrenic acid	36.386	331.26	1.90648	0.000783902	1.22716	0.295322114	up
L-Norleucine	296.72	130.09	2.03609	0.000117789	1.36481	0.448701177	up
Arachidonic acid	37.059	303.23	1.72308	0.004899842	1.24085	0.311328954	up
Undecanoic Acid	46.087	185.15	1.59972	0.040550041	0.60357	-0.72840973	down
3-Methyl-2-oxopentanoate	58.165	129.06	1.4806	0.02273404	1.2983	0.376620548	up
L-Proline	330.62	114.06	1.36091	0.048898098	1.39468	0.479934535	up
4-Dodecylbenzenesulfonic Acid	657.83	325.18	1.38252	0.03792168	1.09922	0.136478949	up
cis,cis-Muconic acid	557.95	141.02	1.46201	0.037351607	0.70919	-0.49576092	down
Thymidine	84.524	241.08	1.92031	0.001345186	1.33029	0.411742025	up
Myristoleic acid	43.775	225.19	1.365	0.024513987	1.2896	0.366924525	up
Eicosapentaenoic acid	37.514	301.22	1.55717	0.016394194	1.33357	0.415292566	up
Phenylpyruvic Acid	56.726	163.04	1.72902	0.00224129	1.34241	0.424827279	up
D-Serine	411.79	104.04	1.82661	0.001651207	1.64584	0.718821711	up
Guanine	244.95	150.04	1.87453	0.000755481	1.38179	0.466535824	up
Carnosine	446.12	225.1	1.98559	0.000516207	1.75837	0.814240912	up
Deoxyguanosine	247.5	266.09	1.59146	0.01400508	1.1946	0.256521881	up
UDP-N-acetyl-alpha-D-galactosamine	423.48	606.07	1.53395	0.020890589	1.37996	0.464628066	up
Pseudouridine	254.96	243.06	1.55717	0.018872807	1.23591	0.305569988	up
L-Glutamine	410.96	145.06	2.05495	0.000323403	1.82326	0.866522573	up
N-Acetyl-L-glutamate	381.53	188.06	1.67291	0.006428817	1.32091	0.401527287	up
N-Acetyl-L-alanine	257.2	130.05	1.8482	0.000883867	1.40302	0.488540705	up
Succinic Acid	382.28	117.02	1.62198	0.011774371	1.29329	0.371044538	up
L-Phenylalanine	280.65	164.07	1.74689	0.002392731	1.43777	0.523830845	up
2-Oxovaleric acid	63.065	115.04	1.59597	0.007766208	1.26093	0.334482692	up
2-Ketoglutaric Acid	365.76	145.01	1.60457	0.016933336	1.45877	0.544752528	up
Uracil	75.791	111.02	2.09533	2.77382E-05	1.42552	0.511489565	up
Hypoxanthine	175.7	135.03	1.93193	0.000425562	1.2253	0.293134122	up
Adipic acid	351.11	145.05	1.54437	0.012139632	0.70851	-0.49713517	down
H-THR-PHE-OH	219.28	265.12	1.62709	0.015175704	1.39197	0.477131304	up
N-Acetylglutamine	316.78	187.07	1.94347	0.001366146	2.21194	1.145311518	up
N-Formyl-L-methionine	196.23	176.04	1.60553	0.016164243	1.52222	0.606175879	up
PI(16:0/16:0)	204.32	809.53	1.29468	0.046690923	0.81901	-0.28804251	down

15(S)-HETE	48.515	319.23	1.48241	0.013925087	1.31365	0.393577401	up
Rutin	25.832	609.13	1.4915	0.017011671	0.80542	-0.31218594	down
Limocitrin	227.06	345.06	1.9148	0.000901898	1.24335	0.314234544	up
Purine	81.789	119.03	1.67023	0.008695242	0.69531	-0.52428181	down
LysoPA(18:1(9Z)/0:0)	210	435.25	1.80386	0.003801659	0.62731	-0.67274494	down
Thymine	65.099	125.04	1.81067	0.002026277	1.37634	0.46083858	up
D-Mannose	82.493	179.06	1.27595	0.045739957	0.81264	-0.29931868	down
Pentadecanoic acid	40.057	241.22	1.4976	0.02351433	1.22842	0.296803265	up
Cytidine	256.35	242.08	1.78988	0.002842345	1.29288	0.370591718	up
LysoPA(16:0/0:0)	218.63	409.23	1.52894	0.04374685	0.82057	-0.28529521	down
Guanosine	281.44	282.08	1.45203	0.031178221	1.22212	0.289380855	up
5-Hydroxyindoleacetic acid	56.97	190.05	1.9159	0.000676191	1.60425	0.681898525	up
Citramalic acid	389.62	147.03	1.56405	0.007913084	1.59333	0.672045794	up
Cimifugin	141.31	305.1	1.54026	0.010486491	0.72106	-0.47181396	down
Epigallocatechin gallate	26.946	457.07	2.00215	9.60394E-05	0.52036	-0.94242296	down
Hydantoin-5-propionic acid	218.63	171.04	1.75582	0.004901817	1.42534	0.511303841	up
3-Oxocholic acid	226.06	451.26	1.46233	0.03132982	0.84453	-0.2437744	down
N-Acetyl-L-methionine	202.56	190.05	1.80741	0.001731102	1.56907	0.649910006	up
Resveratrol	101.12	227.07	1.70424	0.009898362	1.31329	0.393184557	up
Deoxyribose 5-phosphate	350.67	213.02	1.90674	0.000643059	1.37485	0.459276915	up
Theaflavin	425.75	563.12	1.73273	0.039751243	2.26791	1.181365783	up
L-Tyrosine	369.31	180.06	1.87132	0.001355589	1.21525	0.281249841	up
2-hydroxy-2-(4-hydroxy-3-methoxyphenyl)acetic acid	233.48	197.05	1.65644	0.009115771	1.29241	0.370063604	up
D-Alanyl-D-alanine	331.96	159.08	1.76389	0.009769312	1.79894	0.847150677	up
Luteolin 7-glucoside	42.424	448.25	1.50061	0.021419254	0.85603	-0.22426729	down
Ribothymidine	139.91	257.08	1.3413	0.049691636	1.30047	0.379035433	up
Deoxyinosine	189.45	251.08	1.77877	0.00406151	1.16344	0.218402863	up
O-Acetylserine	302.82	146.05	2.10655	3.59905E-05	1.58595	0.665347378	up
3-Phenoxybenzoic acid	61.256	213.05	1.76949	0.002692102	0.60843	-0.71683757	down
Diethyl phthalic acid	58.165	221.08	1.68913	0.005131078	1.42341	0.509354313	up
Aminoadipic acid	271.05	160.06	1.85795	0.002122382	1.37553	0.459988159	up
Ethyl oleate	36.62	309.28	1.70738	0.006125471	0.88066	-0.18334638	down
Acetylglycine	318.18	116.04	2.0919	3.06314E-05	1.49483	0.579979111	up
beta-D-Glucosamine	416.26	214.05	1.66484	0.008781528	1.15016	0.201831358	up
Cytarabine	411.83	242.08	1.41145	0.017802928	0.79789	-0.32573522	down
Psoralen	140.96	185.02	1.14002	0.023795631	1.62893	0.703922682	up
Amentoflavone	418.14	537.1	1.56649	0.017198062	1.57419	0.654607741	up
3-Methylcrotonylglycine	58.918	156.07	1.66239	0.004550818	1.74745	0.805252769	up

L-Theanine	298.35	173.09	1.67444	0.010559943	1.47877	0.564396136	up

RT: Retention time; M/Z: mass charge ratio; VIP: Variable Importance for Projection, one indicator reflecting the capability of the variables to explain Y, FC: Fold change; LOG_FC: LOG_Fold change.
